# National Early Warning Score Does Not Accurately Predict Mortality for Patients With Infection Outside the Intensive Care Unit: A Systematic Review and Meta-Analysis

**DOI:** 10.3389/fmed.2021.704358

**Published:** 2021-07-15

**Authors:** Kai Zhang, Xing Zhang, Wenyun Ding, Nanxia Xuan, Baoping Tian, Tiancha Huang, Zhaocai Zhang, Wei Cui, Huaqiong Huang, Gensheng Zhang

**Affiliations:** ^1^Department of Critical Care Medicine, Second Affiliated Hospital, Zhejiang University School of Medicine, Hangzhou, China; ^2^Medical Security Bureau of Yinzhou District, Ningbo, China; ^3^Department of Respiration and Critical Care Medicine, Second Affiliated Hospital, Zhejiang University School of Medicine, Hangzhou, China; ^4^Department of Respiration Medicine, Community Health Service Center, Shanghai, China

**Keywords:** National early warning score, sepsis, infection, meta-analysis, qSOFA

## Abstract

**Background:** The prognostic value of the national early warning score (NEWS) in patients with infections remains controversial. We aimed to evaluate the prognostic accuracy of NEWS for prediction of in-hospital mortality in patients with infections outside the intensive care unit (ICU).

**Methods:** We searched PubMed, Embase, and Scopus for related articles from January 2012 to April 2021. Sensitivity, specificity, and likelihood ratios were pooled by using the bivariate random-effects model. Overall prognostic performance was summarized by using the area under the curve (AUC). We performed subgroup analyses to assess the prognostic accuracy of NEWS in selected populations.

**Results:** A total of 21 studies with 107,008 participants were included. The pooled sensitivity and specificity of NEWS were 0.71 and 0.60. The pooled AUC of NEWS was 0.70, which was similar to quick sequential organ failure assessment (qSOFA, AUC: 0.70) and better than systemic inflammatory response syndrome (SIRS, AUC: 0.60). However, the sensitivity (0.55) and AUC (0.63) of NEWS were poor in elder patients. The NEWS of 5 was more sensitive, which was a better threshold for activating urgent assessment and treatment.

**Conclusions:** The NEWS had good diagnostic accuracy for early prediction of mortality in patients with infections outside the ICU, and the sensitivity and specificity were more moderate when compared with qSOFA and SIRS. Insufficient sensitivity and poor performance in the elder population may have limitations as an early warning score for adverse outcomes. NEWS should be used for continuous monitoring rather than a single time point predictive tool.

## Introduction

Sepsis, a life-threatening organ dysfunction due to a dysregulated host response to infection, is a major global health problem (1, 2). According to the latest Global Burden of Diseases study, despite declining incidence and mortality, approximately 48.9 million incident cases of sepsis were reported worldwide in 2017, and 11.0 million patients died from sepsis and its complications, accounting for 19.7% of all global deaths (3). Because rapid treatment could improve outcomes of sepsis patients, early identification and risk assessment are of vital importance in the management of sepsis (4, 5). Unfortunately, sepsis remains a complex syndrome with significant heterogeneity and diversity (6); both risk stratification and identification of high-risk patients are still difficult.

The National Early Warning Score (NEWS), first introduced in 2012 and updated in 2017 (National Early Warning Score 2, NEWS2), has received formal endorsement from the National Health Service to become the early warning system for identifying acutely ill patients in the United Kingdom (7, 8). The NEWS is a physiological composite score comprising respiratory rate, oxygen saturation, temperature, blood pressure, pulse rate, and level of consciousness. Each indicator is given a score: 0 is considered normal, and simple addition allows a total score between 0 and 18 to be calculated. A NEWS ≥5 represents the key threshold for urgent response, and patients with NEWS of 7 or more would be deemed to have high clinical risk and trigger a high-level clinical alert (Supplementary File 1). In the guidelines on the recognition, diagnosis and management of sepsis (9), NEWS was recommended for early detection of patients who need more urgent assessment. Moreover, in 2016, the quick sequential organ failure assessment (qSOFA) score was created with the sepsis-3 definitions as a screening tool to identify patients with suspected infection who are likely to have poor outcomes (1). Some studies found that the NEWS was superior to the qSOFA for predicting death or intensive care unit (ICU) admission for patients with infection (10). Although some systematic reviews suggest that NEWS could identify critically ill patients and predict clinically important outcomes (11, 12), the evidence relating to patients with infection is limited (13).

Therefore, we conducted the present study to investigate the prognostic value of NEWS for early prediction of in-hospital mortality in patients with infections outside the ICU. In addition, the performance of the NEWS was compared with that of the qSOFA and systemic inflammatory response syndrome (SIRS) (14) as well.

## Methods

### Study Selection

We followed the guidelines of PRISMA (Supplementary File 2) to structure the meta-analysis (15). A predefined protocol has been registered in PROSPERO (CRD42020164072). We searched PubMed, Embase, and Scopus from January 2012 to April 2021 for relevant articles. The detailed search strategies are reported in Supplementary File 3.

The basic inclusive criteria are as follows: (1) recruited adult patients with infection outside the ICU, (2) applied NEWS to predict in-hospital mortality or 28/30-day mortality, (3) provided sufficient data to estimate the prognostic accuracy. Detailed criteria are recorded in Supplementary File 3.

### Data Extraction

Two authors (X.Z. and W.D.) independently retrieved and extracted studies according to inclusion criteria. We recorded the true positive, false positive, false negative, and true negative from articles directly or through converting of the sensitivity and specificity. Any disagreement in the process was resolved by discussion.

The primary outcome was to estimate the prognostic performance of NEWS for predicting in-hospital mortality. If a study did not report in-hospital mortality, we chose the 28- or 30-day mortality instead. If a study reported the prognostic data on multiple threshold values for the NEWS, we first chose the optimal threshold value (based on the Youden index) for analyses, and then we chose the data on threshold value of ≥5 and ≥7 for subgroup analyses. For included studies that also reported the prognostic performance of qSOFA and/or SIRS (the threshold value for qSOFA and SIRS was ≥2), we estimated the prognostic performance for qSOFA and SIRS to make a comparison with the NEWS. In addition, we also estimated the prognostic performance of NEWS for predicting ICU admission.

### Quality Assessment

Two authors (X.Z. and W.D.) independently employed the PROBAST to assess the risk of bias and applicability concerns of included studies (16). The detailed quality assessment criteria are recorded in Supplementary File 3.

### Statistical Synthesis and Analysis

The bivariate random-effects regression model was employed to pool the sensitivity, specificity, positive likelihood ratio, negative likelihood ratio, and area under the curve (AUC) as point estimate with 95% confidence interval (CI). We also constructed the hierarchical summary receiver operating characteristic (HSROC) curve to present the summary point estimates of sensitivity and specificity. We calculated the *I*^2^ statistics to assess the statistical heterogeneity between included studies, where *I*^2^ > 50% indicated significant heterogeneity (17). We performed subgroup analyses to evaluate the performance of NEWS in selected populations. Studies were stratified according to the age (<70 years old vs. ≥70 years old), disease (non-septic infection vs. sepsis), severity (mortality <10 vs. ≥10%), threshold (≥5 vs. ≥7), setting (emergency department vs. general hospital ward), and study location (United Kingdom vs. other countries). Sensitivity analyses were conducted by repeating the analyses within studies calculating the NEWS at admission.

Publication bias was evaluated by using Deek's test for funnel plot asymmetry (18). All analyses were performed using Stata 12.0 (StataCorp LP, College Station, TX, USA) and Review Manager 5.3 (The Cochrane Collaboration).

## Results

### Study Selection and Characteristics

**Figure 1:**
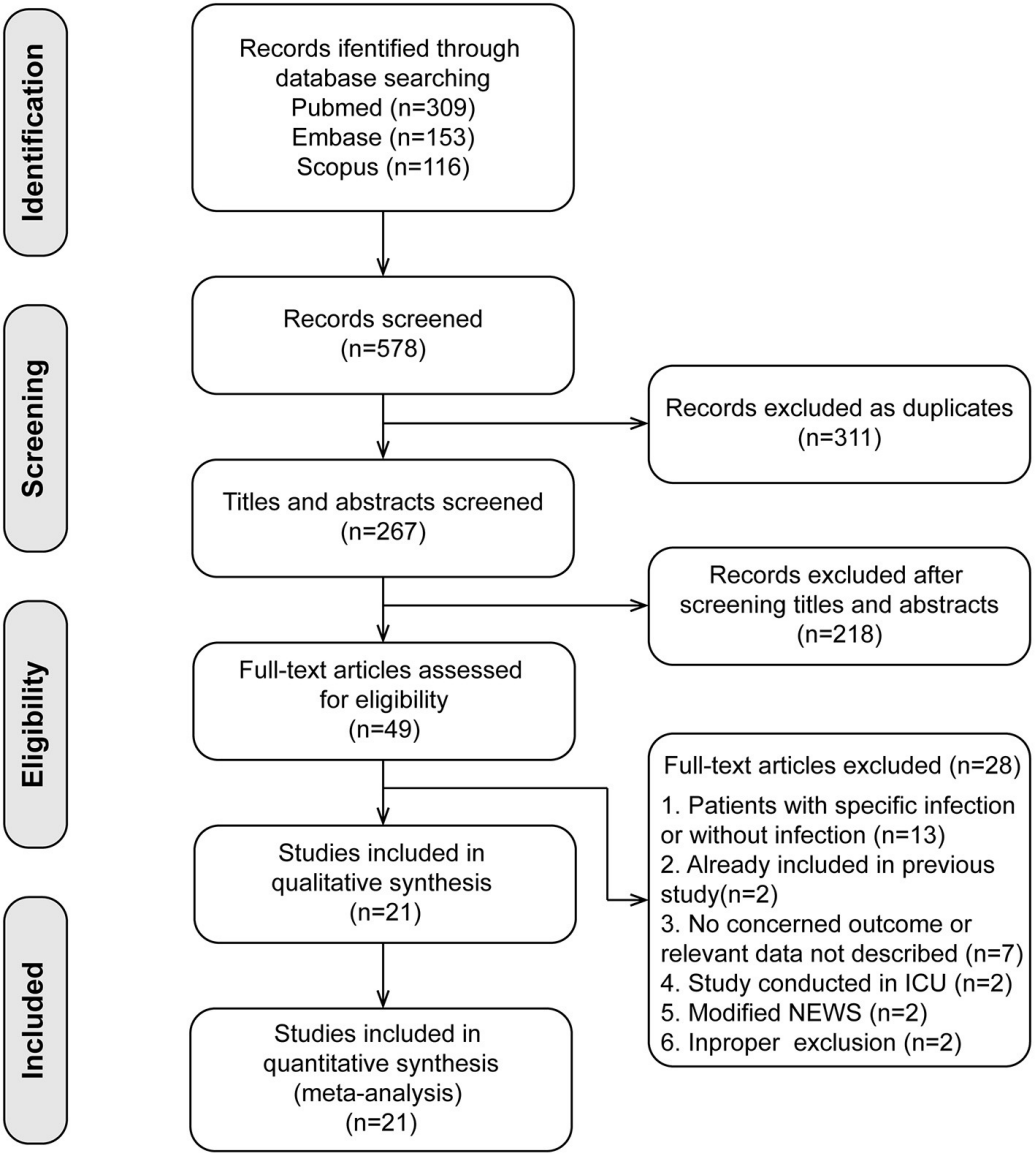
Flow diagram of study inclusion.

A total of 578 published studies were initially identified. After removing duplicate articles and screening abstracts, we identified 49 studies, and 28 studies were excluded with reasons in the full-text assessments. Finally, we included 21 studies (19–39) in our meta-analyses (Figure 1).

Table 1 shows the characteristics of included studies. These studies recruited a total of 107,008 participants, and the mortality rate in each study ranged from 2.5 to 32.8%. Five studies (20, 24, 27, 31, 32) were relatively small in sample size (<400), and 10 studies (19, 23, 26, 28–30, 33, 36, 37, 40) enrolled more than 1,000 patients. Ten studies (19, 21, 22, 27–29, 32–35) included patients with suspected infection, and others focused on patients with suspected sepsis. Six studies investigated general ward patients (23, 29, 31–33, 39), and others evaluated emergency patients. Three studies (21, 23, 31) used the NEWS2, an updated version of NEWS, and others still used the original NEWS. In addition, 16 studies (20, 21, 24–29, 31–37, 39) estimated the prognostic performance of qSOFA, 10 studies (25–27, 29, 32, 34–37, 39) estimated the prognostic performance of SIRS, and five studies (26, 30, 34, 37, 38) also estimated the prognostic performance of NEWS for predicting the ICU admission. Other relevant information is recorded in Supplementary File 4.

**Table 1:** Characteristics of included studies.

**References**	**Simple size**	**Design**	**Population and setting**	**Mortality, *N* (%)**	**Threshold**	**Outcome**
Oduncu et al. (34)	463	Prospective	Suspected infection, in ED	84 (18.1)	NEWS ≥ 5	30-day mortality, ICU admission
Almutary et al. (38)	444	Retrospective	Suspected sepsis, in ED	127 (28.6)	NEWS ≥ 8	In-hospital mortality, ICU admission
Pairattanakorn et al. (39)	409	Prospective	Suspected sepsis, in hospital	117 (28.6)	NEWS ≥ 5	In-hospital mortality
Phungoen et al. (37)	8,177	Retrospective	Suspected sepsis, in ED	509 (6.2)	NEWS ≥ 6	In-hospital mortality, ICU admission
Ruangsomboon et al. (36)	1,622	Retrospective	Suspected sepsis, in ED	457 (28.2)	NEWS ≥ 8	In-hospital mortality
Wattanasit and Khwannimit (35)	777	Retrospective	Suspected infection, in ED	30 (3.9)	NEWS ≥ 7	In-hospital mortality
Saeed et al. (19)	1,175	Retrospective	Suspected infection, in ED	84 (7.1)	NEWS ≥ 7	28-day mortality
Pong et al. (20)	364	Retrospective	Suspected sepsis, in ED	70 (19.2)	NEWS ≥ 8	30-day mortality
Mellhammar et al. (21)	526	Retrospective	Suspected infection, in ED	13 (2.5)	NEWS2 ≥ 5	30-day mortality
Fernando et al. (23)	1,708	Retrospective	Suspected sepsis and assessed by rapid response team, in hospital	560 (32.8)	NEWS2 ≥ 5	In-hospital mortality
Chiew et al. (24)	214	Retrospective	Suspected sepsis, in ED	40 (18.7)	NEWS ≥ 7	30-day mortality
Castille et al. (22)	684	Prospective	Suspected infection, in ED	35 (5.1)	NEWS ≥ 5	In-hospital mortality
Brink et al. (25)	8,204	Retrospective	Suspected sepsis, in ED	490 (6.0)	NEWS ≥ 7	30-day mortality
Ye Lynn et al. (31)	120	Prospective	Sepsis, in hospital	34 (28.3)	NEWS2 ≥ 7	In-hospital mortality
Szakmany et al. (32)	380	Prospective	Infection, in hospital	78 (20.5)	NEWS ≥ 6	30-day mortality
Redfern et al. (33)	44,647	Retrospective	Infection, in hospital	3,035 (6.8)	NEWS ≥ 5	In-hospital mortality
Camm et al. (27)	316	Retrospective	Suspected infection, in ED	25 (7.9)	NEWS ≥ 5	30-day mortality
de Groot et al. (28)	2,280	Retrospective	Suspected infection, in ED	143 (6.3)	NEWS ≥ 8	In-hospital mortality
Goulden et al. (26)	1,818	Retrospective	Suspected sepsis, in ED	265 (15)	NEWS ≥ 5	In-hospital mortality, ICU admission
Churpek et al. (29)	30,677	Retrospective	Suspected infection, in hospital	1,649 (5)	NEWS ≥ 7	In-hospital mortality
Corfield et al. (30)	2,003	Retrospective	Suspected sepsis, in ED	297 (14.8)	NEWS ≥ 7	30-day mortality, ICU admission

*ED, Emergency Department; ICU, Intensive Care Unit; NEWS, National Early Warning Score*.

### Quality Assessment

Table 2 shows the summary results of quality assessments by using PROBAST. Overall, 15 studies had high or unclear risk of bias, mainly because of the inappropriate handling method of missing data (nine studies excluded participants with missing values from analyses; four studies did not explicitly state the handling method of missing data). Seven studies had high or unclear concern regarding applicability because the time interval between the evaluation of the predictor and the determination of the outcome were not consistent with other studies. In addition, Fernando et al. (23) enrolled patients activating a rapid response team, and Pairattanakorn et al. (39) analyzed all hospitalized patients, including some ICU patients. These two studies were rated as high risk of bias and high concern regarding applicability in selection of participants. The details of quality assessment are reported in Supplementary File 5.

**Table 2:** PROBAST results.

**References**	**ROB**				**Applicability**			**Overall**	
	**Participants**	**Predictors**	**Outcome**	**Analysis**	**Participants**	**Predictors**	**Outcome**	**ROB**	**Applicability**
Oduncu et al. (34)	+	+	+	+	+	+	+	+	+
Almutary et al. (38)	?	+	+	?	+	+	+	?	+
Pairattanakorn et al. (39)	–	+	?	–	–	+	?	–	–
Phungoen et al. (37)	–	+	+	–	+	+	+	–	+
Ruangsomboon et al. (36)	+	+	+	+	+	+	+	+	+
Wattanasit and Khwannimit (35)	–	+	+	+	+	+	+	–	+
Saeed et al. (19)	+	+	+	–	+	+	+	–	+
Pong et al. (20)	+	+	+	+	+	+	+	+	+
Mellhammar et al. (21)	+	+	+	–	+	+	+	–	+
Fernando et al. (23)	–	+	+	–	–	+	+	–	–
Chiew et al. (24)	+	+	+	?	+	+	+	?	+
Castille et al. (22)	+	+	+	+	+	+	+	+	+
Brink et al. (25)	+	+	?	+	+	+	?	?	?
Ye Lynn et al. (31)	+	+	+	?	+	+	+	?	+
Szakmany et al. (32)	+	+	+	+	+	+	+	+	+
Redfern et al. (33)	–	+	?	?	+	+	?	–	?
Camm et al. (27)	+	+	?	–	+	+	?	–	?
de Groot et al. (28)	+	+	+	–	+	+	+	–	+
Goulden et al. (26)	+	+	+	+	+	+	+	+	+
Churpek et al. (29)	+	+	?	–	+	+	?	–	?
Corfield et al. (30)	+	+	?	–	+	+	?	–	?

*PROBAST, Prediction model Risk Of Bias ASsessment Tool; ROB, risk of bias. “+” indicates low ROB/low concern regarding applicability; “–” indicates high ROB/high concern regarding applicability; and “?” indicates unclear ROB/unclear concern regarding applicability*.

Furthermore, Deek's funnel plot indicated there was no significant publication bias (Supplementary File 6).

### Results of Synthesis

**Figure 2:**
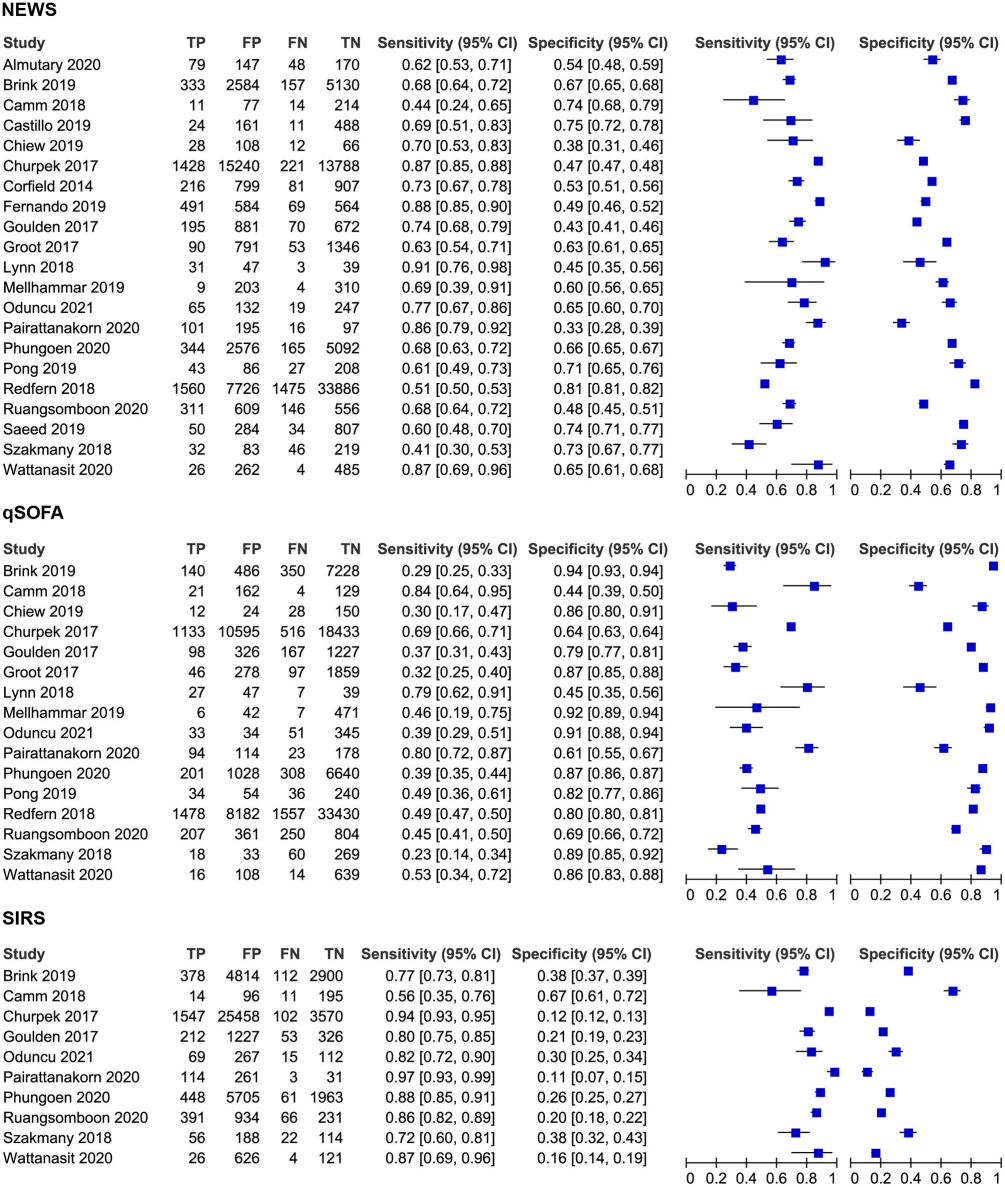
Forest plots of sensitivity and specificity for NEWS, qSOFA and SIRS.

**Figure 3:**
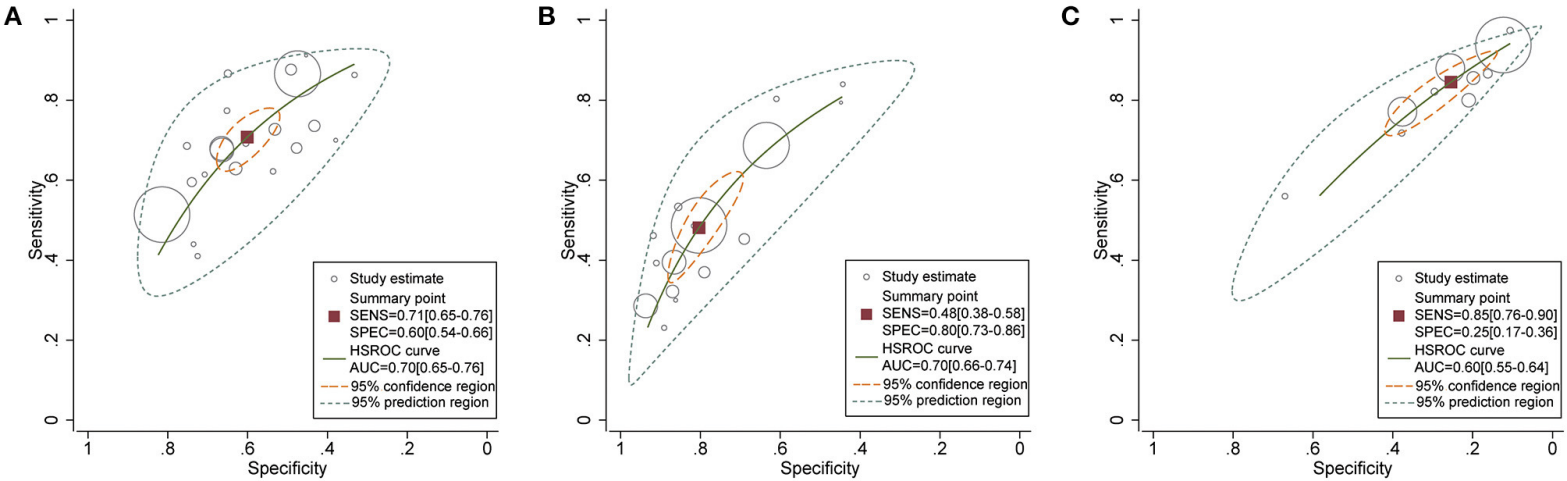
Hierarchical summary ROC curves for **(A)** NEWS, **(B)** qSOFA, **(C)** SIRS for predicting mortality for patients with infection outside the ICU.

Figure 2 shows the forest plot of sensitivity and specificity for NEWS, qSOFA, and SIRS. Figure 3 shows the HSROC curves for NEWS, qSOFA, and SIRS. The pooled sensitivity, specificity, and AUC of NEWS were 0.71 (95%CI 0.65, 0.76), 0.60 (95%CI 0.54, 0.66), and 0.70 (95%CI 0.65, 0.76), respectively. For qSOFA, the pooled sensitivity, specificity, and AUC were 0.48 (95%CI 0.38, 0.58), 0.80 (95%CI 0.73, 0.86), and 0.70 (95%CI 0.66, 0.74), respectively. For SIRS, the pooled sensitivity, specificity, and AUC were 0.85 (95%CI 0.76, 0.90), 0.25 (95%CI 0.17, 0.36), and 0.60 (95%CI 0.55, 0.64), respectively. In addition, the pooled sensitivity, specificity, and AUC of NEWS for predicting the ICU admission were 0.71 (95%CI 0.66, 0.76), 0.55 (95%CI 0.43, 0.65), and 0.71 (95%CI 0.67, 0.75).

### Subgroup and Sensitivity Analyses

There was evidence that prognostic performance varied across different subgroups (Table 3). First, in the cohort of older patients (≥70 years old), the sensitivity was poor (0.55), and the AUC was significant lower than that in younger patients (AUC: 0.63 vs. 0.72, *Z* = 3.562, *P* < 0.001). Second, in patients with sepsis or more severe conditions (mortality rate ≥10%), the NEWS was more sensitive but relatively less specific in predicting mortality. For studies conducted in the United Kingdom, the NEWS was less sensitive (sensitivity: 0.62 vs. 0.75) and the AUC was lower although this was not statistically significant (AUC: 0.68 vs. 0.71, *Z* = 0.653, *P* = 0.514). Moreover, in the 12 studies that used the threshold of 5 or more, the pooled sensitivity, specificity, and AUC were 0.80 (95%CI 0.71, 0.86), 0.50 (95%CI 0.36, 0.63), and 0.73 (95%CI 0.69, 0.76). In nine studies that used the threshold of 7 or more, the pooled sensitivity, specificity, and AUC were 0.74 (95%CI 0.66, 0.81), 0.57 (95%CI 0.47, 0.66), and 0.71 (95%CI 0.67, 0.75).

**Table 3:** Results of meta-analysis.

**Results**	***N***	**Sensitivity (95% CI)**	**Specificity (95% CI)**	**PLR (95% CI)**	**NLR (95% CI)**	**AUC (95% CI)**
NEWS	21	0.71 (0.65, 0.76)	0.60 (0.54, 0.66)	1.77 (1.59, 1.98)	0.49 (0.42, 0.57)	0.70 (0.65, 0.76)
qSOFA	16	0.48 (0.38, 0.58)	0.80 (0.73, 0.86)	2.45 (2.03, 2.96)	0.65 (0.57, 0.74)	0.70 (0.66, 0.74)
SIRS	10	0.85 (0.76, 0.90)	0.25 (0.17, 0.36)	1.13 (1.07, 1.20)	0.61 (0.51, 0.72)	0.60 (0.55, 0.64)
NEWS in predicting ICU admission	5	0.71 (0.66, 0.76)	0.55 (0.43, 0.65)	1.57 (1.29, 1.92)	0.53 (0.46, 0.60)	0.71 (0.67, 0.75)
**Subgroup analysis**						
**Age**						
<70 years old	16	0.72 (0.66, 0.77)	0.61 (0.53, 0.67)	1.83 (1.60, 2.10)	0.46 (0.40, 0.53)	0.72 (0.68, 0.76)
≥70 years old	5	0.55 (0.41, 0.67)	0.64 (0.54, 0.72)	1.50 (1.32, 1.70)	0.71 (0.60, 0.85)	0.63 (0.58, 0.67)
**Threshold value**						
NEWS ≥ 5	12	0.80 (0.71, 0.86)	0.50 (0.36, 0.63)	1.58 (1.30, 1.92)	0.41 (0.34, 0.50)	0.73 (0.69, 0.76)
NEWS ≥ 7	9	0.74 (0.66, 0.81)	0.57 (0.47, 0.66)	1.73 (1.45, 2.06)	0.45 (0.36, 0.57)	0.71 (0.67, 0.75)
**Severity**						
Light	10	0.68 (0.61, 0.75)	0.68 (0.62, 0.73)	2.12 (1.92, 2.34)	0.47 (0.41, 0.55)	0.73 (0.69, 0.77)
Severe	11	0.74 (0.65, 0.81)	0.52 (0.45, 0.60)	1.54 (1.36, 1.74)	0.51 (0.40, 0.64)	0.66 (0.62, 0.70)
**Disease**						
Sepsis	11	0.75 (0.68, 0.80)	0.52 (0.45, 0.59)	1.55 (1.37, 1.75)	0.49 (0.41, 0.59)	0.68 (0.64, 0.72)
Non-septic infection	10	0.66 (0.56, 0.75)	0.69 (0.62, 0.74)	2.11 (1.87, 2.38)	0.49 (0.39, 0.62)	0.73 (0.69, 0.76)
**Setting**						
Emergency department	15	0.68 (0.65, 0.70)	0.62 (0.56, 0.67)	1.77 (1.55, 2.02)	0.52 (0.48, 0.57)	0.70 (0.65, 0.73)
General ward	6	0.78 (0.62, 0.89)	0.56 (0.41, 0.70)	1.80 (1.47, 2.20)	0.38 (0.26, 0.57)	0.72 (0.68, 0.76)
**Location**						
UK	6	0.62 (0.56, 0.69)	0.68 (0.56, 0.78)	1.92 (1.53, 2.42)	0.56 (0.53, 0.60)	0.68 (0.64, 0.72)
Other countries	15	0.75 (0.69, 0.80)	0.57 (0.51, 0.63)	1.75 (1.55, 1.98)	0.43 (0.36, 0.53)	0.71 (0.67, 0.75)
**Sensitivity analysis**						
At admission	15	0.70 (0.63, 0.77)	0.60 (0.54, 0.66)	1.76 (1.54, 2.01)	0.49 (0.41, 0.60)	0.70 (0.65, 0.73)
Excluded studies with small sample size	16	0.73 (0.67, 0.78)	0.60 (0.53, 0.66)	1.80 (1.59, 2.04)	0.46 (0.38, 0.54)	0.71 (0.67, 0.75)

*NEWS, National Early Warning Score; qSOFA, quick Sequential Organ Failure Assessment; SIRS, Systemic Inflammatory Response Syndrome; ICU, Intensive Care Unit; N, Number of studies; CI, Confidence Interval; PLR, Positive Likelihood Ratio; NLR, Negative Likelihood Ratio; AUC, Area Under the Curve*.

In addition, when we restricted analysis to studies (19–24, 26, 28, 31, 32, 34–38) that evaluated the NEWS at admission or excluded five studies with small sample sizes (20, 24, 27, 31, 32), the sensitivity analyses showed similar results to the primary result.

## Discussion

In this study, we analyzed the prognostic accuracy of the NEWS for mortality in patients with infections outside the ICU and compared the performance with that of the qSOFA and SIRS. We found the NEWS had good diagnostic accuracy for early prediction of mortality in patients with infections outside the ICU, and the sensitivity and specificity were moderate when compared with qSOFA and SIRS. That the NEWS ≥5 was more sensitive for predicting mortality in patients with infections outside the ICU indicates that 5 points is an optimal threshold for activating urgent treatment or critical care input.

Estimates of pooled results showed considerable heterogeneity between studies. Investigating the source of heterogeneity and the prognostic performance of NEWS in selected populations are important objectives in our study. First, aging appears to decrease the predictive accuracy of NEWS. Our results indicate that the NEWS could not accurately predict mortality in the elder population. Due to the relatively low sensitivity and AUC, the NEWS was of limited prognostic value in elder patients with infection. Second, the severity of disease might affect the prognostic accuracy. Our study population was composed of two groups: patients with non-septic infection and patients with sepsis. Applying the NEWS in septic patients who have higher mortality rates might result in greater predictive probabilities of death as opposed to employing it among patients with non-septic infection. The subgroup analysis indicated that the sensitivity of NEWS in the sepsis subgroup was higher than the non-septic infection subgroup. Besides this, different ways to identify infection (e.g., patients with positive blood cultures or who received intravenous antibiotics) or sepsis (e.g., sepsis-1 or sepsis-3 criteria) may have been responsible for heterogeneity. Furthermore, study location might be a source of heterogeneity because differences in the health care systems of each country could affect clinical outcomes. Specifically, early warning score systems have been introduced and linked to effective clinical responses in many UK hospitals (41). It might introduce the treatment paradox, by which some deteriorating patients were likely to receive rapid medical interventions after triggering the alert. Hence, the actual mortality tends to be lower than predicted and biases our estimate of accuracy. In addition, our study included patients with different infection types, and variations in outcome measures (in-hospital or 28/30-day mortality) could also account for heterogeneity.

### Comparisons With Previous Literature

Sepsis is a common cause of adverse clinical outcomes among patients with infections, and acute management is the foundation of improved outcomes for these patients (5). The main problem in the management of sepsis outside the ICU is identification of high-risk patients since the first assessment. This way, early treatments can be started, which would alter the septic patient's prognosis. Clinicians previously used the SIRS criteria to diagnose sepsis for patients with infection, which is criticized because the criteria were too non-specific (42). The sepsis-3 definitions task force developed the qSOFA score as an early warning risk stratification tool for identification and escalation of care in septic patients outside the ICU (43). However, Song et al. investigate the prognostic value of qSOFA and SIRS in patients with infection outside the ICU and indicate that qSOFA could not serve as a predictive tool for adverse outcomes because of its low sensitivity (0.51) (44). Similarly, Fernando et al. comprehensively investigate the prognostic accuracy of qSOFA in different populations and find that qSOFA had significant lower sensitivity in patients outside the ICU than others in the ICU (0.46 vs. 0.87) (45). In our study, we prove that the qSOFA score had the highest specificity but low sensitivity in predicted mortality, which was consistent with previous studies (44, 45). Compared with qSOFA and SIRS, the NEWS had moderate sensitivity and specificity and was superior to the qSOFA for predicting mortality.

In addition to the NEWS, qSOFA, and SIRS, some severity scoring systems have been widely used outside the ICU (12). The mortality in emergency department sepsis (MEDS) score was specially developed for emergency patients with suspected infections to predict the 28-day mortality rate (46). Our current study also reveals that MEDS has good discrimination (AUC 0.83) and moderate sensitivity (0.79) and specificity (0.74) (47). Some of the score components rely on a clinician's subjective assessment (e.g., terminal illness, altered mental status), and the neutrophil bands were not routinely measured in some departments. These weaknesses limit the scope of its implementation. The modified early warning score (MEWS) is another frequently used prognostic score system, which is considered the most reliable method to assess in-hospital mortality in the general population (12). However, when applied to patients with sepsis, Hamilton et al. analyzes five trials and suggests the MEWS has poor prognostic value in predicting sepsis mortality (48). In addition, we further searched relevant studies and performed an updated meta-analysis, and our results reconfirmed that MEWS could not accurately identify patients at high risk of death (49).

Furthermore, it is possible that the prognostic accuracy of the NEWS could be improved by combining some important clinical parameters. For instance, research shows that older patients with infection have a higher death rate (3), and increased age is independently associated with poor prognosis in septic patients (50). Thus, a Chinese group put forward a modified version of the NEWS with the addition of age >65 years as an independent component, termed NEWS-C (51). An external validation study found the NEWS-C has the best predictive accuracy among common scoring systems for predicting deterioration of respiratory function in patients with COVID-19 (52). Moreover, lactate is a strong and independent predictor of mortality for patients with infection (53). A modified early warning score combining the NEWS and initial serum lactate level, called NEWS-L, is proved to have good discriminant value for identifying high-risk patients in the emergency department (54).

### Implications for Practice

Although our research suggests that NEWS has moderate prognostic performance, it is worth highlighting some potential pitfalls in clinical practice. First of all, the qSOFA only contains three components and three boundary levels, whereas NEWS has seven components with more than 20 boundaries. Certainly, the application of NEWS requires additional resources and could become a burden on clinicians, especially in situations of overcrowding in the emergency wards. Second, the NEWS is not an ideal screening tool to identify high-risk patients because of its limited sensitivity. It means that some critically ill patients may be improperly classified as non-severe, even delayed treatment, which is devastating to the patients. Therefore, the NEWS is not an alternative to the clinical judgment by experienced clinicians, and it should be utilized to help clinical decision making by providing objective data. Even negative NEWS should not prevent clinicians from conducting further assessment and management of patients with suspected infection, indicating that experienced clinical judgment remains vital (13). Finally, in addition to the initial assessment of illness severity, the NEWS is also recommended to be used as a track and trigger tool to identify acute clinical deterioration and guide the clinical response for patients. By recording the NEWS on a regular basis, the trends in the patient's clinical response can be tracked (7). Therefore, the score should be calculated not only at patient admission but also throughout the hospital stay as part of the standard clinical observation chart to evaluate a possible deterioration in the clinical situation (48).

### Strength and Limitations

Strengths of this meta-analysis include a standard protocol and comprehensive search strategies across multiple databases. Thus, we believe that we did not miss any relevant studies. Second, a statistically robust hierarchical model was employed to estimate pooled results and to construct HSROC plots. This approach allows for both between-study variability in sensitivity and specificity and flexibility in the estimation of summary statistics (55). Our findings can contribute to a better understanding of NEWS in patients with infections outside the ICU and could be useful for implementing NEWS in clinical practice.

Meanwhile, there are some important limitations in the meta-analysis. First, previous research suggests heterogeneities are widely observed in systematic reviews of diagnostic test accuracy (44, 56). We also identify significant heterogeneity among included studies, which might affect the credibility of the pooled estimates. Although we directly compared the NEWS with qSOFA using the same cohort of patients to minimize heterogeneity, the results should be interpreted prudently. Second, both the NEWS and qSOFA were developed for detecting patients with high risk of clinical deterioration rather than predicting in-hospital mortality. This may be the main reason why NEWS shows poor performance in our meta-analysis. Further research should focus on the prognostic accuracy of NEWS for predicting clinical deterioration. Third, as only three studies evaluated the NEWS2, the insufficient data could not develop reliable conclusions regarding the potential benefits of the updated score over the original NEWS. Furthermore, the NEWS was not designed as a single time point predictive tool. Because existing research only shows the prognostic accuracy of NEWS in predicting mortality at a single time point (mostly at the time of admission), we could not evaluate NEWS in any other context. On the other hand, the timing of NEWS measurement was not entirely consistent in included studies. Given the dynamic nature of sepsis, we assume that the accuracy might be improved if multiple time points were considered. The change trend of NEWS with time has potential application value of predicting mortality, just like the delta SOFA (57).

## Conclusion

The NEWS has good diagnostic accuracy for early prediction of mortality in patients with infections outside the ICU, the sensitivity and specificity were more moderate when compared with qSOFA and SIRS. NEWS of 5 or more was an optimal trigger threshold for activating a rapid response. However, as an early warning score, both NEWS and qSOFA had a significant weakness that insufficient sensitivity could delay lifesaving treatment for critical patients. The NEWS should be used for continuous monitoring of patients' condition and guide clinical response, not solely for initial assessment of illness severity. We suggest that developing enhanced or modified scoring systems is quite necessary, and future early warning scores could be devised by using machine learning algorithms.

## Data Availability Statement

The original contributions presented in the study are included in the article/Supplementary Material, further inquiries can be directed to the corresponding author/s.

## Author Contributions

KZ conceived the idea, performed the analysis, and drafted the manuscript. XZ and WD contributed to the study design, data acquisition, and interpretation. NX, BT, and TH helped in the statistical analysis. ZZ and WC critically revised the manuscript for important intellectual content. GZ and HH helped to frame the idea of the study and provided technical support. All authors contributed to the article and approved the submitted version.

## Conflict of Interest

The authors declare that the research was conducted in the absence of any commercial or financial relationships that could be construed as a potential conflict of interest.

## Abbreviations

ICU: Intensive care unit
qSOFA: quick Sequential Organ Failure Assessment
SOFA: Sequential Organ Failure Assessment
AUC: Area Under the Curve
NEWS: National Early Warning Score
CI: confidence interval
PLR: positive likelihood ratio
NLR: negative likelihood ratio
DOR: diagnostic odds ratio
HSROC: hierarchical summary receiver operating characteristic
QUADAS: Quality Assessment of Diagnostic Accuracy Studies.
