# Supplementary material for: National Early Warning Score Does Not Accurately Predict Mortality for Patients With Infection Outside the Intensive Care Unit: A Systematic Review and Meta-Analysis
Source: Front Med (Lausanne). 2021 Jul 15;8:704358. doi: 10.3389/fmed.2021.704358 (PMC8319382; doi:10.3389/fmed.2021.704358)
Supplement: Supplementary file 1 [file Data_Sheet_1.pdf]

Chart 1: National Early Warning Score (NEWS)*

PHYSIOLOGICAL PARAMETERS	3	2	1	0	1	2	3
Respiration Rate	≤8		9 - 11	12 - 20		21 - 24	≥25
Oxygen Saturations	≤91	92 - 93	94 - 95	≥96			
Any Supplemental Oxygen		Yes		No			
Temperature	≤35.0		35.1 - 36.0	36.1 - 38.0	38.1 - 39.0	≥39.1	
Systolic BP	≤90	91 - 100	101 - 110	111 - 219			≥220
Heart Rate	≤40		41 - 50	51 - 90	91 - 110	111 - 130	≥131
Level of Consciousness				A			V, P, or U

*The NEWS initiative flowed from the Royal College of Physicians' NEWSDIG, and was jointly developed and funded in collaboration with the Royal College of Physicians, Royal College of Nursing, National Outreach Forum and NHS Training for Innovation.

Royal College of Physicians

NHS Training for Innovation

Chart 2: The NEWS scoring system (NEWS2)

Physiological parameter				Score			
	3	2	1	0	1	2	3
Respiration rate (per minute)	≤8		9–11	12–20		21–24	≥25
SpO_2 Scale 1 (%)	≤91	92–93	94–95	≥96			
SpO_2 Scale 2 (%)	≤83	84–85	86–87	88–92 ≥93 on air	93–94 on oxygen	95–96 on oxygen	≥97 on oxygen
Air or oxygen?		Oxygen		Air			
Systolic blood pressure (mmHg)	≤90	91–100	101–110	111–219			≥220
Pulse (per minute)	≤40		41–50	51–90	91–110	111–130	≥131
Consciousness				Alert			CVPU
Temperature (°C)	≤35.0		35.1–36.0	36.1–38.0	38.1–39.0	≥39.1	

Chart 3: NEWS thresholds and triggers

NEW score	Clinical risk	Response
Aggregate score 0–4	Low	Ward-based response
Red score Score of 3 in any individual parameter	Low–medium	Urgent ward-based response*
Aggregate score 5–6	Medium	Key threshold for urgent response*
Aggregate score 7 or more	High	Urgent or emergency response**

* Response by a clinician or team with competence in the assessment and treatment of acutely ill patients and in recognising when the escalation of care to a critical care team is appropriate.

**The response team must also include staff with critical care skills, including airway management.
