# Supplementary material for: National Early Warning Score Does Not Accurately Predict Mortality for Patients With Infection Outside the Intensive Care Unit: A Systematic Review and Meta-Analysis
Source: Front Med (Lausanne). 2021 Jul 15;8:704358. doi: 10.3389/fmed.2021.704358 (PMC8319382; doi:10.3389/fmed.2021.704358)
Supplement: Supplementary file 3 [file Data_Sheet_3.docx]

**Supplementary File 3:** Searching strategies, inclusion and exclusion criteria, and quality assessment criteria

**Searching strategies**

**Pubmed**

#1 sepsis[Title/Abstract] OR septic[Title/Abstract] OR sepsis[MeSH Terms] OR septic shock[MeSH Terms] OR infection[Title/Abstract] OR infection[MeSH Terms] OR infect*[Title/Abstract]

#2 mortality[Title/Abstract] OR mortality[MeSH Terms] OR death[Title/Abstract] OR death[MeSH Terms]

#3 news [Title/Abstract] OR national early warning score [Title/Abstract]

#1 AND #2 AND #3

**Embase**

#1 sepsis:ab,ti OR infection:ab,ti OR septic:ab,ti OR 'sepsis'/exp OR 'infection'/exp

#2 mortality:ab,ti OR 'mortality'/exp

#3 (national early warning score):ab,ti

#1 AND #2 AND #3

**Scopus**

#1 TITLE-ABS-KEY (sepsis) OR TITLE-ABS-KEY (septic) OR TITLE-ABS-KEY (infection) OR TITLE-ABS-KEY (infect)

#2 TITLE-ABS-KEY (mortality) OR TITLE-ABS-KEY (death)

#3 TITLE-ABS-KEY (national early warning score)

#1 AND #2 AND #3

**Inclusion criteria**

(1) The study recruited adult patients (≥18 years old) with infection or sepsis outside the ICU;

(2) The study applied the NEWS for predicting mortality (28-day mortality, 30-day mortality or in-hospital mortality);

(3) The study should provide sufficient data to calculate the true positive (TP), false positive (FP), false negative (FN), true negative (TN) results;

(4) Full-length articles written in English and research object was limited to human;

**Exclusion criteria**

1. The study population included non-adult or uninfected patients;
2. The study included specified patients (e.g. community acquired pneumonia, coronavirus disease 2019)
3. The article was not written in English;
4. The study did not report sufficient data to calculate TP, FP, FN, and TN results;
5. Case reports, case series, animal studies, pediatric studies;
6. The study evaluated NEWS only for a composite outcome (e.p. combination of in-hospital mortality, ICU admission, adverse outcomes and so on);
7. If studies used the same database, we included the study with the most patients and excluded the others

**Quality Assessment Criteria**

Two authors independently assessed the risk of bias of included trials by using the Prediction model Risk Of Bias ASsessment Tool (PROBAST). The PROBAST consists assessment of four key domains to judge the quality of studies: participants, predictors, outcome, analysis. The answer to each item was “+”, “-” or “?” (“+” indicates low risk of bias; “-” indicates high risk of bias; and “?” indicates unclear risk of bias). If a study was judged as “low” on all domains relating to bias, then it was assigned an overall judgment of “low risk of bias” or “low concern regarding applicability”, and had high quality. If a study was judged “high” in one or more domains, then it may have been judged as “at risk of bias” or “concerns regarding applicability”. Disagreements were resolved by third author.
