# Supplementary material for: National Early Warning Score Does Not Accurately Predict Mortality for Patients With Infection Outside the Intensive Care Unit: A Systematic Review and Meta-Analysis
Source: Front Med (Lausanne). 2021 Jul 15;8:704358. doi: 10.3389/fmed.2021.704358 (PMC8319382; doi:10.3389/fmed.2021.704358)
Supplement: Supplementary file 4 [file Data_Sheet_4.pdf]

Corfield et al.

DOMAIN 1: Participants		
**A. Risk of Bias**		
1.1 Were appropriate data sources used, e.g. cohort, RCT or nested case-control study data?		Yes
1.2 Were all inclusions and exclusions of participants appropriate?		Yes
**Risk of bias introduced by selection of participants**	**RISK:** (low/ high/ unclear)	Low
Rationale of bias rating: Low risk of bias		
**B. Applicability**		
**Concern that the included participants and setting do not match the review question**	**CONCERN:** (low/ high/ unclear)	Low
Rationale of applicability rating: Match the review question		

DOMAIN 2: Predictors		
**A. Risk of Bias**		
2.1 Were predictors defined and assessed in a similar way for all participants?		Yes
2.2 Were predictor assessments made without knowledge of outcome data?		Yes
2.3 Are all predictors available at the time the model is intended to be used?		Yes
**Risk of bias introduced by predictors or their assessment**	**RISK:** (low/ high/ unclear)	Low
Rationale of bias rating: Low risk of bias		
**B. Applicability**		
Concern that the definition, assessment or timing of predictors in the model do not match the review question	**CONCERN:** (low/ high/ unclear)	Low
Rationale of applicability rating: Match the review question		

DOMAIN 3: Outcome		
**A. Risk of Bias**		
3.1 Was the outcome determined appropriately?		Yes
3.2 Was a pre-specified or standard outcome definition used?		Yes
3.3 Were predictors excluded from the outcome definition?		Yes
3.4 Was the outcome defined and determined in a similar way for all participants?		Yes
3.5 Was the outcome determined without knowledge of predictor information?		Yes
3.6 Was the time interval between predictor assessment and outcome determination appropriate?		No information
**Risk of bias introduced by the outcome or its determination**	**RISK:** (low/ high/ unclear)	Unclear
Rationale of bias rating: Did not report the time of calculating score		
**B. Applicability**		
**Concern that the outcome, its definition, timing or determination do not match the review question**	**CONCERN:** (low/ high/ unclear)	Unclear
Rationale of applicability rating: Did not report the time of calculating score		

DOMAIN 4: Analysis		
**Risk of Bias**		
4.1 Were there a reasonable number of participants with the outcome?		Yes
4.2 Were continuous and categorical predictors handled appropriately?		Yes
4.3 Were all enrolled participants included in the analysis?		Yes
4.4 Were participants with missing data handled appropriately?		No
4.5 Was selection of predictors based on univariable analysis avoided?		Yes
4.6 Were complexities in the data (e.g. censoring, competing risks, sampling of controls) accounted for appropriately?		Yes
4.7 Were relevant model performance measures evaluated appropriately?		Yes
4.8 Were model overfitting and optimism in model performance accounted for?		Not applicable
4.9 Do predictors and their assigned weights in the final model correspond to the results from multivariable analysis?		Not applicable
**Risk of bias introduced by the analysis**	**RISK:** (low/ high/ unclear)	Low
Rationale of bias rating: Patients with missing value were excluded		

Churpek et al.

DOMAIN 1: Participants		
**A. Risk of Bias**		
1.1 Were appropriate data sources used, e.g. cohort, RCT or nested case-control study data?		Yes
1.2 Were all inclusions and exclusions of participants appropriate?		Yes
**Risk of bias introduced by selection of participants**	**RISK:** (low/ high/ unclear)	Low
Rationale of bias rating: Low risk of bias		
**B. Applicability**		
**Concern that the included participants and setting do not match the review question**	**CONCERN:** (low/ high/ unclear)	Low
Rationale of applicability rating: Match the review question		

DOMAIN 2: Predictors		
**A. Risk of Bias**		
2.1 Were predictors defined and assessed in a similar way for all participants?		Yes
2.2 Were predictor assessments made without knowledge of outcome data?		Yes
2.3 Are all predictors available at the time the model is intended to be used?		Yes
**Risk of bias introduced by predictors or their assessment**	**RISK:** (low/ high/ unclear)	Low
Rationale of bias rating: Low risk of bias		
**B. Applicability**		
Concern that the definition, assessment or timing of predictors in the model do not match the review question	**CONCERN:** (low/ high/ unclear)	Low
Rationale of applicability rating: Match the review question		

DOMAIN 3: Outcome		
**A. Risk of Bias**		
3.1 Was the outcome determined appropriately?		Yes
3.2 Was a pre-specified or standard outcome definition used?		Yes
3.3 Were predictors excluded from the outcome definition?		Yes
3.4 Was the outcome defined and determined in a similar way for all participants?		Yes
3.5 Was the outcome determined without knowledge of predictor information?		Yes
3.6 Was the time interval between predictor assessment and outcome determination appropriate?		No information
**Risk of bias introduced by the outcome or its determination**	**RISK:** (low/ high/ unclear)	Unclear
Rationale of bias rating: Did not report the time of calculating score		
**B. Applicability**		
**Concern that the outcome, its definition, timing or determination do not match the review question**	**CONCERN:** (low/ high/ unclear)	Unclear
Rationale of applicability rating: Did not report the time of calculating score		

DOMAIN 4: Analysis		
**Risk of Bias**		
4.1 Were there a reasonable number of participants with the outcome?		Yes
4.2 Were continuous and categorical predictors handled appropriately?		Yes
4.3 Were all enrolled participants included in the analysis?		Yes
4.4 Were participants with missing data handled appropriately?		No
4.5 Was selection of predictors based on univariable analysis avoided?		Yes
4.6 Were complexities in the data (e.g. censoring, competing risks, sampling of controls) accounted for appropriately?		Yes
4.7 Were relevant model performance measures evaluated appropriately?		Yes
4.8 Were model overfitting and optimism in model performance accounted for?		Not applicable
4.9 Do predictors and their assigned weights in the final model correspond to the results from multivariable analysis?		Not applicable
**Risk of bias introduced by the analysis**	**RISK:** (low/ high/ unclear)	Low
Rationale of bias rating: Previous values were pulled forward if they were missing, and if no previous values were available, a median value was imputed		

Goulden et al.

DOMAIN 1: Participants		
**A. Risk of Bias**		
1.1 Were appropriate data sources used, e.g. cohort, RCT or nested case-control study data?		Yes
1.2 Were all inclusions and exclusions of participants appropriate?		Yes
**Risk of bias introduced by selection of participants**	**RISK:** (low/ high/ unclear)	Low
Rationale of bias rating: Low risk of bias		
**B. Applicability**		
**Concern that the included participants and setting do not match the review question**	**CONCERN:** (low/ high/ unclear)	Low
Rationale of applicability rating: Match the review question		

DOMAIN 2: Predictors		
**A. Risk of Bias**		
2.1 Were predictors defined and assessed in a similar way for all participants?		Yes
2.2 Were predictor assessments made without knowledge of outcome data?		Yes
2.3 Are all predictors available at the time the model is intended to be used?		Yes
**Risk of bias introduced by predictors or their assessment**	**RISK:** (low/ high/ unclear)	Low
Rationale of bias rating: Low risk of bias		
**B. Applicability**		
Concern that the definition, assessment or timing of predictors in the model do not match the review question	**CONCERN:** (low/ high/ unclear)	Low
Rationale of applicability rating: Match the review question		

DOMAIN 3: Outcome		
**A. Risk of Bias**		
3.1 Was the outcome determined appropriately?		Yes
3.2 Was a pre-specified or standard outcome definition used?		Yes
3.3 Were predictors excluded from the outcome definition?		Yes
3.4 Was the outcome defined and determined in a similar way for all participants?		Yes
3.5 Was the outcome determined without knowledge of predictor information?		Yes
3.6 Was the time interval between predictor assessment and outcome determination appropriate?		Yes
**Risk of bias introduced by the outcome or its determination**	**RISK:** (low/ high/ unclear)	Low
Rationale of bias rating: Low risk of bias		
**B. Applicability**		
**Concern that the outcome, its definition, timing or determination do not match the review question**	**CONCERN:** (low/ high/ unclear)	Low
Rationale of applicability rating: Match the review question		

DOMAIN 4: Analysis		
**Risk of Bias**		
4.1 Were there a reasonable number of participants with the outcome?		Yes
4.2 Were continuous and categorical predictors handled appropriately?		Yes
4.3 Were all enrolled participants included in the analysis?		Yes
4.4 Were participants with missing data handled appropriately?		Yes
4.5 Was selection of predictors based on univariable analysis avoided?		Yes
4.6 Were complexities in the data (e.g. censoring, competing risks, sampling of controls) accounted for appropriately?		Yes
4.7 Were relevant model performance measures evaluated appropriately?		Yes
4.8 Were model overfitting and optimism in model performance accounted for?		Not applicable
4.9 Do predictors and their assigned weights in the final model correspond to the results from multivariable analysis?		Not applicable
**Risk of bias introduced by the analysis**	**RISK:** (low/ high/ unclear)	Low
Rationale of bias rating: Low risk of bias		

Groot et al.

DOMAIN 1: Participants		
**A. Risk of Bias**		
1.1 Were appropriate data sources used, e.g. cohort, RCT or nested case-control study data?		Yes
1.2 Were all inclusions and exclusions of participants appropriate?		Yes
**Risk of bias introduced by selection of participants**	**RISK:** (low/ high/ unclear)	Low
Rationale of bias rating: Low risk of bias		
**B. Applicability**		
**Concern that the included participants and setting do not match the review question**	**CONCERN:** (low/ high/ unclear)	Low
Rationale of applicability rating: Match the review question		

DOMAIN 2: Predictors		
**A. Risk of Bias**		
2.1 Were predictors defined and assessed in a similar way for all participants?		Yes
2.2 Were predictor assessments made without knowledge of outcome data?		Yes
2.3 Are all predictors available at the time the model is intended to be used?		Yes
**Risk of bias introduced by predictors or their assessment**	**RISK:** (low/ high/ unclear)	Low
Rationale of bias rating: Low risk of bias		
**B. Applicability**		
Concern that the definition, assessment or timing of predictors in the model do not match the review question	**CONCERN:** (low/ high/ unclear)	Low
Rationale of applicability rating: Match the review question		

DOMAIN 3: Outcome		
**A. Risk of Bias**		
3.1 Was the outcome determined appropriately?		Yes
3.2 Was a pre-specified or standard outcome definition used?		Yes
3.3 Were predictors excluded from the outcome definition?		Yes
3.4 Was the outcome defined and determined in a similar way for all participants?		Yes
3.5 Was the outcome determined without knowledge of predictor information?		Yes
3.6 Was the time interval between predictor assessment and outcome determination appropriate?		Yes
**Risk of bias introduced by the outcome or its determination**	**RISK:** (low/ high/ unclear)	Low
Rationale of bias rating: Low risk of bias		
**B. Applicability**		
**Concern that the outcome, its definition, timing or determination do not match the review question**	**CONCERN:** (low/ high/ unclear)	Low
Rationale of applicability rating: Match the review question		

DOMAIN 4: Analysis		
**Risk of Bias**		
4.1 Were there a reasonable number of participants with the outcome?		Yes
4.2 Were continuous and categorical predictors handled appropriately?		Yes
4.3 Were all enrolled participants included in the analysis?		Yes
4.4 Were participants with missing data handled appropriately?		No
4.5 Was selection of predictors based on univariable analysis avoided?		Yes
4.6 Were complexities in the data (e.g. censoring, competing risks, sampling of controls) accounted for appropriately?		Yes
4.7 Were relevant model performance measures evaluated appropriately?		Yes
4.8 Were model overfitting and optimism in model performance accounted for?		Not applicable
4.9 Do predictors and their assigned weights in the final model correspond to the results from multivariable analysis?		Not applicable
**Risk of bias introduced by the analysis**	**RISK:** (low/ high/ unclear)	High
Rationale of bias rating:<br>Missing data were assumed to be normal		

Camm et al.

DOMAIN 1: Participants		
**A. Risk of Bias**		
1.1 Were appropriate data sources used, e.g. cohort, RCT or nested case-control study data?		Yes
1.2 Were all inclusions and exclusions of participants appropriate?		Yes
**Risk of bias introduced by selection of participants**	**RISK:** (low/ high/ unclear)	Low
Rationale of bias rating: Low risk of bias		
**B. Applicability**		
**Concern that the included participants and setting do not match the review question**	**CONCERN:** (low/ high/ unclear)	Low
Rationale of applicability rating: Match the review question		

DOMAIN 2: Predictors		
**A. Risk of Bias**		
2.1 Were predictors defined and assessed in a similar way for all participants?		Yes
2.2 Were predictor assessments made without knowledge of outcome data?		Yes
2.3 Are all predictors available at the time the model is intended to be used?		Yes
**Risk of bias introduced by predictors or their assessment**	**RISK:** (low/ high/ unclear)	Low
Rationale of bias rating: Low risk of bias		
**B. Applicability**		
Concern that the definition, assessment or timing of predictors in the model do not match the review question	**CONCERN:** (low/ high/ unclear)	Low
Rationale of applicability rating: Match the review question		

DOMAIN 3: Outcome		
**A. Risk of Bias**		
3.1 Was the outcome determined appropriately?		Yes
3.2 Was a pre-specified or standard outcome definition used?		Yes
3.3 Were predictors excluded from the outcome definition?		Yes
3.4 Was the outcome defined and determined in a similar way for all participants?		Yes
3.5 Was the outcome determined without knowledge of predictor information?		Yes
3.6 Was the time interval between predictor assessment and outcome determination appropriate?		Unclear
**Risk of bias introduced by the outcome or its determination**	**RISK:** (low/ high/ unclear)	Unclear
Rationale of bias rating: Did not report the time of calculating scores		
**B. Applicability**		
**Concern that the outcome, its definition, timing or determination do not match the review question**	**CONCERN:** (low/ high/ unclear)	Unclear
Rationale of applicability rating: Did not report the time of calculating scores		

DOMAIN 4: Analysis		
**Risk of Bias**		
4.1 Were there a reasonable number of participants with the outcome?		Yes
4.2 Were continuous and categorical predictors handled appropriately?		Yes
4.3 Were all enrolled participants included in the analysis?		Yes
4.4 Were participants with missing data handled appropriately?		No
4.5 Was selection of predictors based on univariable analysis avoided?		Yes
4.6 Were complexities in the data (e.g. censoring, competing risks, sampling of controls) accounted for appropriately?		Yes
4.7 Were relevant model performance measures evaluated appropriately?		Yes
4.8 Were model overfitting and optimism in model performance accounted for?		Not applicable
4.9 Do predictors and their assigned weights in the final model correspond to the results from multivariable analysis?		Not applicable
**Risk of bias introduced by the analysis**	**RISK:** (low/ high/ unclear)	High
Rationale of bias rating: Patients with missing data were excluded		

Lynn et al.

DOMAIN 1: Participants		
**A. Risk of Bias**		
1.1 Were appropriate data sources used, e.g. cohort, RCT or nested case-control study data?		Yes
1.2 Were all inclusions and exclusions of participants appropriate?		Yes
**Risk of bias introduced by selection of participants**	**RISK:** (low/ high/ unclear)	Low
Rationale of bias rating: Low risk of bias		
**B. Applicability**		
**Concern that the included participants and setting do not match the review question**	**CONCERN:** (low/ high/ unclear)	Low
Rationale of applicability rating: Match the review question		

DOMAIN 2: Predictors		
**A. Risk of Bias**		
2.1 Were predictors defined and assessed in a similar way for all participants?		Yes
2.2 Were predictor assessments made without knowledge of outcome data?		Yes
2.3 Are all predictors available at the time the model is intended to be used?		Yes
**Risk of bias introduced by predictors or their assessment**	**RISK:** (low/ high/ unclear)	Low
Rationale of bias rating: Low risk of bias		
**B. Applicability**		
Concern that the definition, assessment or timing of predictors in the model do not match the review question	**CONCERN:** (low/ high/ unclear)	Low
Rationale of applicability rating: Match the review question		

DOMAIN 3: Outcome		
**A. Risk of Bias**		
3.1 Was the outcome determined appropriately?		Yes
3.2 Was a pre-specified or standard outcome definition used?		Yes
3.3 Were predictors excluded from the outcome definition?		Yes
3.4 Was the outcome defined and determined in a similar way for all participants?		Yes
3.5 Was the outcome determined without knowledge of predictor information?		Yes
3.6 Was the time interval between predictor assessment and outcome determination appropriate?		Yes
**Risk of bias introduced by the outcome or its determination**	**RISK:** (low/ high/ unclear)	Low
Rationale of bias rating: Low risk of bias		
**B. Applicability**		
**Concern that the outcome, its definition, timing or determination do not match the review question**	**CONCERN:** (low/ high/ unclear)	Low
Rationale of applicability rating: Match the review question		

DOMAIN 4: Analysis		
**Risk of Bias**		
4.1 Were there a reasonable number of participants with the outcome?		Yes
4.2 Were continuous and categorical predictors handled appropriately?		Yes
4.3 Were all enrolled participants included in the analysis?		Yes
4.4 Were participants with missing data handled appropriately?		No information
4.5 Was selection of predictors based on univariable analysis avoided?		Yes
4.6 Were complexities in the data (e.g. censoring, competing risks, sampling of controls) accounted for appropriately?		Yes
4.7 Were relevant model performance measures evaluated appropriately?		Yes
4.8 Were model overfitting and optimism in model performance accounted for?		Not applicable
4.9 Do predictors and their assigned weights in the final model correspond to the results from multivariable analysis?		Not applicable
**Risk of bias introduced by the analysis**	**RISK:** (low/ high/ unclear)	Unclear
Rationale of bias rating: Did not report the missing data		

Brink et al.

DOMAIN 1: Participants		
**A. Risk of Bias**		
1.1 Were appropriate data sources used, e.g. cohort, RCT or nested case-control study data?		No
1.2 Were all inclusions and exclusions of participants appropriate?		Yes
**Risk of bias introduced by selection of participants**	**RISK:** (low/ high/ unclear)	High
Rationale of bias rating:<br>It is a retrospective cohort study		
**B. Applicability**		
**Concern that the included participants and setting do not match the review question**	**CONCERN:** (low/ high/ unclear)	Low
Rationale of applicability rating:<br>Match the review question		

DOMAIN 2: Predictors		
**A. Risk of Bias**		
2.1 Were predictors defined and assessed in a similar way for all participants?		Yes
2.2 Were predictor assessments made without knowledge of outcome data?		Yes
2.3 Are all predictors available at the time the model is intended to be used?		Yes
**Risk of bias introduced by predictors or their assessment**	**RISK:** (low/ high/ unclear)	Low
Rationale of bias rating:<br>Low risk of bias		
**B. Applicability**		
Concern that the definition, assessment or timing of predictors in the model do not match the review question	**CONCERN:** (low/ high/ unclear)	Low
Rationale of applicability rating:<br>Match the review question		

DOMAIN 3: Outcome		
**A. Risk of Bias**		
3.1 Was the outcome determined appropriately?		Yes
3.2 Was a pre-specified or standard outcome definition used?		Yes
3.3 Were predictors excluded from the outcome definition?		Yes
3.4 Was the outcome defined and determined in a similar way for all participants?		Yes
3.5 Was the outcome determined without knowledge of predictor information?		Yes
3.6 Was the time interval between predictor assessment and outcome determination appropriate?		No information
**Risk of bias introduced by the outcome or its determination**	**RISK:** (low/ high/ unclear)	Unclear
Rationale of bias rating:<br>Did not report the time of calculating score		
**B. Applicability**		
**Concern that the outcome, its definition, timing or determination do not match the review question**	**CONCERN:** (low/ high/ unclear)	Unclear
Rationale of applicability rating:<br>Did not report the time of calculating score		

DOMAIN 4: Analysis		
**Risk of Bias**		
4.1 Were there a reasonable number of participants with the outcome?		Yes
4.2 Were continuous and categorical predictors handled appropriately?		Yes
4.3 Were all enrolled participants included in the analysis?		Yes
4.4 Were participants with missing data handled appropriately?		No information
4.5 Was selection of predictors based on univariable analysis avoided?		Yes
4.6 Were complexities in the data (e.g. censoring, competing risks, sampling of controls) accounted for appropriately?		Yes
4.7 Were relevant model performance measures evaluated appropriately?		Yes
4.8 Were model overfitting and optimism in model performance accounted for?		Not applicable
4.9 Do predictors and their assigned weights in the final model correspond to the results from multivariable analysis?		Not applicable
**Risk of bias introduced by the analysis**	**RISK:** (low/ high/ unclear)	Unclear
Rationale of bias rating: Did not report the missing data		

Szakmany et al.

DOMAIN 1: Participants		
**A. Risk of Bias**		
1.1 Were appropriate data sources used, e.g. cohort, RCT or nested case-control study data?		Yes
1.2 Were all inclusions and exclusions of participants appropriate?		Yes
**Risk of bias introduced by selection of participants**	**RISK:** (low/ high/ unclear)	Low
Rationale of bias rating:<br>Low risk of bias		
**B. Applicability**		
**Concern that the included participants and setting do not match the review question**	**CONCERN:** (low/ high/ unclear)	Low
Rationale of applicability rating:<br>Match the review question		

DOMAIN 2: Predictors		
**A. Risk of Bias**		
2.1 Were predictors defined and assessed in a similar way for all participants?		Yes
2.2 Were predictor assessments made without knowledge of outcome data?		Yes
2.3 Are all predictors available at the time the model is intended to be used?		Yes
**Risk of bias introduced by predictors or their assessment**	**RISK:** (low/ high/ unclear)	Low
Rationale of bias rating:<br>Low risk of bias		
**B. Applicability**		
Concern that the definition, assessment or timing of predictors in the model do not match the review question	**CONCERN:** (low/ high/ unclear)	Low
Rationale of applicability rating:<br>Match the review question		

DOMAIN 3: Outcome		
**A. Risk of Bias**		
3.1 Was the outcome determined appropriately?		Yes
3.2 Was a pre-specified or standard outcome definition used?		Yes
3.3 Were predictors excluded from the outcome definition?		Yes
3.4 Was the outcome defined and determined in a similar way for all participants?		Yes
3.5 Was the outcome determined without knowledge of predictor information?		Yes
3.6 Was the time interval between predictor assessment and outcome determination appropriate?		Yes
**Risk of bias introduced by the outcome or its determination**	**RISK:** (low/ high/ unclear)	Low
Rationale of bias rating:<br>Low risk of bias		
**B. Applicability**		
**Concern that the outcome, its definition, timing or determination do not match the review question**	**CONCERN:** (low/ high/ unclear)	Low
Rationale of applicability rating:<br>Match the review question		

DOMAIN 4: Analysis		
**Risk of Bias**		
4.1 Were there a reasonable number of participants with the outcome?		Yes
4.2 Were continuous and categorical predictors handled appropriately?		Yes
4.3 Were all enrolled participants included in the analysis?		Yes
4.4 Were participants with missing data handled appropriately?		Yes
4.5 Was selection of predictors based on univariable analysis avoided?		Yes
4.6 Were complexities in the data (e.g. censoring, competing risks, sampling of controls) accounted for appropriately?		Yes
4.7 Were relevant model performance measures evaluated appropriately?		Yes
4.8 Were model overfitting and optimism in model performance accounted for?		Not applicable
4.9 Do predictors and their assigned weights in the final model correspond to the results from multivariable analysis?		Not applicable
**Risk of bias introduced by the analysis**	**RISK:** (low/ high/ unclear)	Low
Rationale of bias rating:<br>Low risk of bias		

Brink et al.

DOMAIN 1: Participants		
**A. Risk of Bias**		
1.1 Were appropriate data sources used, e.g. cohort, RCT or nested case-control study data?		Yes
1.2 Were all inclusions and exclusions of participants appropriate?		Yes
**Risk of bias introduced by selection of participants**	**RISK:** (low/ high/ unclear)	Low
Rationale of bias rating: Low risk of bias		
**B. Applicability**		
**Concern that the included participants and setting do not match the review question**	**CONCERN:** (low/ high/ unclear)	Low
Rationale of applicability rating: Match the review question		

DOMAIN 2: Predictors		
**A. Risk of Bias**		
2.1 Were predictors defined and assessed in a similar way for all participants?		Yes
2.2 Were predictor assessments made without knowledge of outcome data?		Yes
2.3 Are all predictors available at the time the model is intended to be used?		Yes
**Risk of bias introduced by predictors or their assessment**	**RISK:** (low/ high/ unclear)	Low
Rationale of bias rating: Low risk of bias		
**B. Applicability**		
Concern that the definition, assessment or timing of predictors in the model do not match the review question	**CONCERN:** (low/ high/ unclear)	Low
Rationale of applicability rating: Match the review question		

DOMAIN 3: Outcome		
**A. Risk of Bias**		
3.1 Was the outcome determined appropriately?		Yes
3.2 Was a pre-specified or standard outcome definition used?		Yes
3.3 Were predictors excluded from the outcome definition?		Yes
3.4 Was the outcome defined and determined in a similar way for all participants?		Yes
3.5 Was the outcome determined without knowledge of predictor information?		Yes
3.6 Was the time interval between predictor assessment and outcome determination appropriate?		No information
**Risk of bias introduced by the outcome or its determination**	**RISK:** (low/ high/ unclear)	Unclear
Rationale of bias rating: Did not report the time of calculating score		
**B. Applicability**		
**Concern that the outcome, its definition, timing or determination do not match the review question**	**CONCERN:** (low/ high/ unclear)	Unclear
Rationale of applicability rating: Did not report the time of calculating score		

DOMAIN 4: Analysis		
**Risk of Bias**		
4.1 Were there a reasonable number of participants with the outcome?		Yes
4.2 Were continuous and categorical predictors handled appropriately?		Yes
4.3 Were all enrolled participants included in the analysis?		Yes
4.4 Were participants with missing data handled appropriately?		Yes
4.5 Was selection of predictors based on univariable analysis avoided?		Yes
4.6 Were complexities in the data (e.g. censoring, competing risks, sampling of controls) accounted for appropriately?		Yes
4.7 Were relevant model performance measures evaluated appropriately?		Yes
4.8 Were model overfitting and optimism in model performance accounted for?		Not applicable
4.9 Do predictors and their assigned weights in the final model correspond to the results from multivariable analysis?		Not applicable
**Risk of bias introduced by the analysis**	**RISK:** (low/ high/ unclear)	Low
Rationale of bias rating: Low risk of bias		

Castillo et al.

DOMAIN 1: Participants		
**A. Risk of Bias**		
1.1 Were appropriate data sources used, e.g. cohort, RCT or nested case-control study data?		Yes
1.2 Were all inclusions and exclusions of participants appropriate?		Yes
**Risk of bias introduced by selection of participants**	**RISK:** (low/ high/ unclear)	Low
Rationale of bias rating: Low risk of bias		
**B. Applicability**		
**Concern that the included participants and setting do not match the review question**	**CONCERN:** (low/ high/ unclear)	Low
Rationale of applicability rating: Match the review question		

DOMAIN 2: Predictors		
**A. Risk of Bias**		
2.1 Were predictors defined and assessed in a similar way for all participants?		Yes
2.2 Were predictor assessments made without knowledge of outcome data?		Yes
2.3 Are all predictors available at the time the model is intended to be used?		Yes
**Risk of bias introduced by predictors or their assessment**	**RISK:** (low/ high/ unclear)	Low
Rationale of bias rating: Low risk of bias		
**B. Applicability**		
Concern that the definition, assessment or timing of predictors in the model do not match the review question	**CONCERN:** (low/ high/ unclear)	Low
Rationale of applicability rating: Match the review question		

DOMAIN 3: Outcome		
**A. Risk of Bias**		
3.1 Was the outcome determined appropriately?		Yes
3.2 Was a pre-specified or standard outcome definition used?		Yes
3.3 Were predictors excluded from the outcome definition?		Yes
3.4 Was the outcome defined and determined in a similar way for all participants?		Yes
3.5 Was the outcome determined without knowledge of predictor information?		Yes
3.6 Was the time interval between predictor assessment and outcome determination appropriate?		Yes
**Risk of bias introduced by the outcome or its determination**	**RISK:** (low/ high/ unclear)	Low
Rationale of bias rating: Low risk of bias		
**B. Applicability**		
**Concern that the outcome, its definition, timing or determination do not match the review question**	**CONCERN:** (low/ high/ unclear)	Low
Rationale of applicability rating: Match the review question		

DOMAIN 4: Analysis		
**Risk of Bias**		
4.1 Were there a reasonable number of participants with the outcome?		Yes
4.2 Were continuous and categorical predictors handled appropriately?		Yes
4.3 Were all enrolled participants included in the analysis?		Yes
4.4 Were participants with missing data handled appropriately?		Yes
4.5 Was selection of predictors based on univariable analysis avoided?		Yes
4.6 Were complexities in the data (e.g. censoring, competing risks, sampling of controls) accounted for appropriately?		Yes
4.7 Were relevant model performance measures evaluated appropriately?		Yes
4.8 Were model overfitting and optimism in model performance accounted for?		Not applicable
4.9 Do predictors and their assigned weights in the final model correspond to the results from multivariable analysis?		Not applicable
**Risk of bias introduced by the analysis**	**RISK:** (low/ high/ unclear)	Low
Rationale of bias rating:<br>Low risk of bias		

Chiew et al.

DOMAIN 1: Participants		
**A. Risk of Bias**		
1.1 Were appropriate data sources used, e.g. cohort, RCT or nested case-control study data?		Yes
1.2 Were all inclusions and exclusions of participants appropriate?		Yes
**Risk of bias introduced by selection of participants**	**RISK:** (low/ high/ unclear)	Low
Rationale of bias rating: Low risk of bias		
**B. Applicability**		
**Concern that the included participants and setting do not match the review question**	**CONCERN:** (low/ high/ unclear)	Low
Rationale of applicability rating: Match the review question		

DOMAIN 2: Predictors		
**A. Risk of Bias**		
2.1 Were predictors defined and assessed in a similar way for all participants?		Yes
2.2 Were predictor assessments made without knowledge of outcome data?		Yes
2.3 Are all predictors available at the time the model is intended to be used?		Yes
**Risk of bias introduced by predictors or their assessment**	**RISK:** (low/ high/ unclear)	Low
Rationale of bias rating: Low risk of bias		
**B. Applicability**		
Concern that the definition, assessment or timing of predictors in the model do not match the review question	**CONCERN:** (low/ high/ unclear)	Low
Rationale of applicability rating: Match the review question		

DOMAIN 3: Outcome		
**A. Risk of Bias**		
3.1 Was the outcome determined appropriately?		Yes
3.2 Was a pre-specified or standard outcome definition used?		Yes
3.3 Were predictors excluded from the outcome definition?		Yes
3.4 Was the outcome defined and determined in a similar way for all participants?		Yes
3.5 Was the outcome determined without knowledge of predictor information?		Yes
3.6 Was the time interval between predictor assessment and outcome determination appropriate?		Yes
**Risk of bias introduced by the outcome or its determination**	**RISK:** (low/ high/ unclear)	Low
Rationale of bias rating: Low risk of bias		
**B. Applicability**		
**Concern that the outcome, its definition, timing or determination do not match the review question**	**CONCERN:** (low/ high/ unclear)	Low
Rationale of applicability rating: Match the review question		

DOMAIN 4: Analysis		
**Risk of Bias**		
4.1 Were there a reasonable number of participants with the outcome?		Yes
4.2 Were continuous and categorical predictors handled appropriately?		Yes
4.3 Were all enrolled participants included in the analysis?		Yes
4.4 Were participants with missing data handled appropriately?		No information
4.5 Was selection of predictors based on univariable analysis avoided?		Yes
4.6 Were complexities in the data (e.g. censoring, competing risks, sampling of controls) accounted for appropriately?		Yes
4.7 Were relevant model performance measures evaluated appropriately?		Yes
4.8 Were model overfitting and optimism in model performance accounted for?		Not applicable
4.9 Do predictors and their assigned weights in the final model correspond to the results from multivariable analysis?		Not applicable
**Risk of bias introduced by the analysis**	**RISK:** (low/ high/ unclear)	Unclear
Rationale of bias rating:<br>Did not report the missing data		

Fernando et al.

DOMAIN 1: Participants		
**A. Risk of Bias**		
1.1 Were appropriate data sources used, e.g. cohort, RCT or nested case-control study data?		Yes
1.2 Were all inclusions and exclusions of participants appropriate?		No
**Risk of bias introduced by selection of participants**	**RISK:** (low/ high/ unclear)	High
Rationale of bias rating:<br>Included patients fulfilled the rapid response team calling criteria		
**B. Applicability**		
**Concern that the included participants and setting do not match the review question**	**CONCERN:** (low/ high/ unclear)	High
Rationale of applicability rating:<br>Included patients fulfilled the rapid response team calling criteria		

DOMAIN 2: Predictors		
**A. Risk of Bias**		
2.1 Were predictors defined and assessed in a similar way for all participants?		Yes
2.2 Were predictor assessments made without knowledge of outcome data?		Yes
2.3 Are all predictors available at the time the model is intended to be used?		Yes
**Risk of bias introduced by predictors or their assessment**	**RISK:** (low/ high/ unclear)	Low
Rationale of bias rating:<br>Low risk of bias		
**B. Applicability**		
Concern that the definition, assessment or timing of predictors in the model do not match the review question	**CONCERN:** (low/ high/ unclear)	Low
Rationale of applicability rating:<br>Match the review question		

DOMAIN 3: Outcome		
**A. Risk of Bias**		
3.1 Was the outcome determined appropriately?		Yes
3.2 Was a pre-specified or standard outcome definition used?		Yes
3.3 Were predictors excluded from the outcome definition?		Yes
3.4 Was the outcome defined and determined in a similar way for all participants?		Yes
3.5 Was the outcome determined without knowledge of predictor information?		Yes
3.6 Was the time interval between predictor assessment and outcome determination appropriate?		Yes
**Risk of bias introduced by the outcome or its determination**	**RISK:** (low/ high/ unclear)	Low
Rationale of bias rating:<br>Low risk of bias		
**B. Applicability**		
**Concern that the outcome, its definition, timing or determination do not match the review question**	**CONCERN:** (low/ high/ unclear)	Low
Rationale of applicability rating:<br>Match the review question		

DOMAIN 4: Analysis		
**Risk of Bias**		
4.1 Were there a reasonable number of participants with the outcome?		Yes
4.2 Were continuous and categorical predictors handled appropriately?		Yes
4.3 Were all enrolled participants included in the analysis?		Yes
4.4 Were participants with missing data handled appropriately?		No
4.5 Was selection of predictors based on univariable analysis avoided?		Yes
4.6 Were complexities in the data (e.g. censoring, competing risks, sampling of controls) accounted for appropriately?		Yes
4.7 Were relevant model performance measures evaluated appropriately?		Yes
4.8 Were model overfitting and optimism in model performance accounted for?		Not applicable
4.9 Do predictors and their assigned weights in the final model correspond to the results from multivariable analysis?		Not applicable
**Risk of bias introduced by the analysis**	**RISK:** (low/ high/ unclear)	High
Rationale of bias rating: Patients with missing value were excluded		

Melhammar et al.

DOMAIN 1: Participants		
**A. Risk of Bias**		
1.1 Were appropriate data sources used, e.g. cohort, RCT or nested case-control study data?		Yes
1.2 Were all inclusions and exclusions of participants appropriate?		Yes
**Risk of bias introduced by selection of participants**	**RISK:** (low/ high/ unclear)	Low
Rationale of bias rating: Low risk of bias		
**B. Applicability**		
**Concern that the included participants and setting do not match the review question**	**CONCERN:** (low/ high/ unclear)	Low
Rationale of applicability rating: Match the review question		

DOMAIN 2: Predictors		
**A. Risk of Bias**		
2.1 Were predictors defined and assessed in a similar way for all participants?		Yes
2.2 Were predictor assessments made without knowledge of outcome data?		Yes
2.3 Are all predictors available at the time the model is intended to be used?		Yes
**Risk of bias introduced by predictors or their assessment**	**RISK:** (low/ high/ unclear)	Low
Rationale of bias rating: Low risk of bias		
**B. Applicability**		
Concern that the definition, assessment or timing of predictors in the model do not match the review question	**CONCERN:** (low/ high/ unclear)	Low
Rationale of applicability rating: Match the review question		

DOMAIN 3: Outcome		
**A. Risk of Bias**		
3.1 Was the outcome determined appropriately?		Yes
3.2 Was a pre-specified or standard outcome definition used?		Yes
3.3 Were predictors excluded from the outcome definition?		Yes
3.4 Was the outcome defined and determined in a similar way for all participants?		Yes
3.5 Was the outcome determined without knowledge of predictor information?		Yes
3.6 Was the time interval between predictor assessment and outcome determination appropriate?		Yes
**Risk of bias introduced by the outcome or its determination**	**RISK:** (low/ high/ unclear)	Low
Rationale of bias rating: Low risk of bias		
**B. Applicability**		
**Concern that the outcome, its definition, timing or determination do not match the review question**	**CONCERN:** (low/ high/ unclear)	Low
Rationale of applicability rating: Match the review question		

DOMAIN 4: Analysis		
**Risk of Bias**		
4.1 Were there a reasonable number of participants with the outcome?		Yes
4.2 Were continuous and categorical predictors handled appropriately?		Yes
4.3 Were all enrolled participants included in the analysis?		Yes
4.4 Were participants with missing data handled appropriately?		No
4.5 Was selection of predictors based on univariable analysis avoided?		Yes
4.6 Were complexities in the data (e.g. censoring, competing risks, sampling of controls) accounted for appropriately?		Yes
4.7 Were relevant model performance measures evaluated appropriately?		Yes
4.8 Were model overfitting and optimism in model performance accounted for?		Not applicable
4.9 Do predictors and their assigned weights in the final model correspond to the results from multivariable analysis?		Not applicable
**Risk of bias introduced by the analysis**	**RISK:** (low/ high/ unclear)	High
Rationale of bias rating:<br>Patients with missing value were excluded		

Pong et al.

DOMAIN 1: Participants		
**A. Risk of Bias**		
1.1 Were appropriate data sources used, e.g. cohort, RCT or nested case-control study data?		Yes
1.2 Were all inclusions and exclusions of participants appropriate?		Yes
**Risk of bias introduced by selection of participants**	**RISK:** (low/ high/ unclear)	Low
Rationale of bias rating: Low risk of bias		
**B. Applicability**		
**Concern that the included participants and setting do not match the review question**	**CONCERN:** (low/ high/ unclear)	Low
Rationale of applicability rating: Match the review question		

DOMAIN 2: Predictors		
**A. Risk of Bias**		
2.1 Were predictors defined and assessed in a similar way for all participants?		Yes
2.2 Were predictor assessments made without knowledge of outcome data?		Yes
2.3 Are all predictors available at the time the model is intended to be used?		Yes
**Risk of bias introduced by predictors or their assessment**	**RISK:** (low/ high/ unclear)	Low
Rationale of bias rating: Low risk of bias		
**B. Applicability**		
Concern that the definition, assessment or timing of predictors in the model do not match the review question	**CONCERN:** (low/ high/ unclear)	Low
Rationale of applicability rating: Match the review question		

DOMAIN 3: Outcome		
**A. Risk of Bias**		
3.1 Was the outcome determined appropriately?		Yes
3.2 Was a pre-specified or standard outcome definition used?		Yes
3.3 Were predictors excluded from the outcome definition?		Yes
3.4 Was the outcome defined and determined in a similar way for all participants?		Yes
3.5 Was the outcome determined without knowledge of predictor information?		Yes
3.6 Was the time interval between predictor assessment and outcome determination appropriate?		Yes
**Risk of bias introduced by the outcome or its determination**	**RISK:** (low/ high/ unclear)	Low
Rationale of bias rating: Low risk of bias		
**B. Applicability**		
**Concern that the outcome, its definition, timing or determination do not match the review question**	**CONCERN:** (low/ high/ unclear)	Low
Rationale of applicability rating: Match the review question		

DOMAIN 4: Analysis		
**Risk of Bias**		
4.1 Were there a reasonable number of participants with the outcome?		Yes
4.2 Were continuous and categorical predictors handled appropriately?		Yes
4.3 Were all enrolled participants included in the analysis?		Yes
4.4 Were participants with missing data handled appropriately?		Yes
4.5 Was selection of predictors based on univariable analysis avoided?		Yes
4.6 Were complexities in the data (e.g. censoring, competing risks, sampling of controls) accounted for appropriately?		Yes
4.7 Were relevant model performance measures evaluated appropriately?		Yes
4.8 Were model overfitting and optimism in model performance accounted for?		Not applicable
4.9 Do predictors and their assigned weights in the final model correspond to the results from multivariable analysis?		Not applicable
**Risk of bias introduced by the analysis**	**RISK:** (low/ high/ unclear)	Low
Rationale of bias rating: Low risk of bias		

Saeed et al.

DOMAIN 1: Participants		
**A. Risk of Bias**		
1.1 Were appropriate data sources used, e.g. cohort, RCT or nested case-control study data?		Yes
1.2 Were all inclusions and exclusions of participants appropriate?		Yes
**Risk of bias introduced by selection of participants**	**RISK:** (low/ high/ unclear)	Low
Rationale of bias rating:<br>Low risk of bias		
**B. Applicability**		
**Concern that the included participants and setting do not match the review question**	**CONCERN:** (low/ high/ unclear)	Low
Rationale of applicability rating:<br>Match the review question		

DOMAIN 2: Predictors		
**A. Risk of Bias**		
2.1 Were predictors defined and assessed in a similar way for all participants?		Yes
2.2 Were predictor assessments made without knowledge of outcome data?		Yes
2.3 Are all predictors available at the time the model is intended to be used?		Yes
**Risk of bias introduced by predictors or their assessment**	**RISK:** (low/ high/ unclear)	Low
Rationale of bias rating:<br>Low risk of bias		
**B. Applicability**		
Concern that the definition, assessment or timing of predictors in the model do not match the review question	**CONCERN:** (low/ high/ unclear)	Low
Rationale of applicability rating:<br>Match the review question		

DOMAIN 3: Outcome		
**A. Risk of Bias**		
3.1 Was the outcome determined appropriately?		Yes
3.2 Was a pre-specified or standard outcome definition used?		Yes
3.3 Were predictors excluded from the outcome definition?		Yes
3.4 Was the outcome defined and determined in a similar way for all participants?		Yes
3.5 Was the outcome determined without knowledge of predictor information?		Yes
3.6 Was the time interval between predictor assessment and outcome determination appropriate?		Yes
**Risk of bias introduced by the outcome or its determination**	**RISK:** (low/ high/ unclear)	Low
Rationale of bias rating:<br>Low risk of bias		
**B. Applicability**		
**Concern that the outcome, its definition, timing or determination do not match the review question**	**CONCERN:** (low/ high/ unclear)	Low
Rationale of applicability rating:<br>Match the review question		

<table>
<tr><th colspan="3">DOMAIN 4: Analysis</th></tr>
<tr><th colspan="3">Risk of Bias</th></tr>
<tr><td colspan="2">4.1 Were there a reasonable number of participants with the outcome?</td><td>Yes</td></tr>
<tr><td colspan="2">4.2 Were continuous and categorical predictors handled appropriately?</td><td>Yes</td></tr>
<tr><td colspan="2">4.3 Were all enrolled participants included in the analysis?</td><td>Yes</td></tr>
<tr><td colspan="2">4.4 Were participants with missing data handled appropriately?</td><td>No</td></tr>
<tr><td colspan="2">4.5 Was selection of predictors based on univariable analysis avoided?</td><td>Yes</td></tr>
<tr><td colspan="2">4.6 Were complexities in the data (e.g. censoring, competing risks, sampling of controls) accounted for appropriately?</td><td>Yes</td></tr>
<tr><td colspan="2">4.7 Were relevant model performance measures evaluated appropriately?</td><td>Yes</td></tr>
<tr><td colspan="2">4.8 Were model overfitting and optimism in model performance accounted for?</td><td>Not applicable</td></tr>
<tr><td colspan="2">4.9 Do predictors and their assigned weights in the final model correspond to the results from multivariable analysis?</td><td>Not applicable</td></tr>
<tr><td>Risk of bias introduced by the analysis</td><td>RISK:
(low/ high/ unclear)</td><td>High</td></tr>
<tr><td colspan="3">Rationale of bias rating:
Patients with missing value were excluded</td></tr>
</table>

Almutary et al.

DOMAIN 1: Participants		
**A. Risk of Bias**		
1.1 Were appropriate data sources used, e.g. cohort, RCT or nested case-control study data?		Yes
1.2 Were all inclusions and exclusions of participants appropriate?		Unclear
**Risk of bias introduced by selection of participants**	**RISK:** (low/ high/ unclear)	Unclear
Rationale of bias rating:<br>Did not report the definition of suspected sepsis		
**B. Applicability**		
**Concern that the included participants and setting do not match the review question**	**CONCERN:** (low/ high/ unclear)	Low
Rationale of applicability rating:<br>Match the review question		

DOMAIN 2: Predictors		
**A. Risk of Bias**		
2.1 Were predictors defined and assessed in a similar way for all participants?		Yes
2.2 Were predictor assessments made without knowledge of outcome data?		Yes
2.3 Are all predictors available at the time the model is intended to be used?		Yes
**Risk of bias introduced by predictors or their assessment**	**RISK:** (low/ high/ unclear)	Low
Rationale of bias rating:<br>Low risk of bias		
**B. Applicability**		
Concern that the definition, assessment or timing of predictors in the model do not match the review question	**CONCERN:** (low/ high/ unclear)	Low
Rationale of applicability rating:<br>Match the review question		

DOMAIN 3: Outcome		
**A. Risk of Bias**		
3.1 Was the outcome determined appropriately?		Yes
3.2 Was a pre-specified or standard outcome definition used?		Yes
3.3 Were predictors excluded from the outcome definition?		Yes
3.4 Was the outcome defined and determined in a similar way for all participants?		Yes
3.5 Was the outcome determined without knowledge of predictor information?		Yes
3.6 Was the time interval between predictor assessment and outcome determination appropriate?		Yes
**Risk of bias introduced by the outcome or its determination**	**RISK:** (low/ high/ unclear)	Low
Rationale of bias rating:<br>Low risk of bias		
**B. Applicability**		
**Concern that the outcome, its definition, timing or determination do not match the review question**	**CONCERN:** (low/ high/ unclear)	Low
Rationale of applicability rating:<br>Match the review question		

DOMAIN 4: Analysis		
**Risk of Bias**		
4.1 Were there a reasonable number of participants with the outcome?		Yes
4.2 Were continuous and categorical predictors handled appropriately?		Yes
4.3 Were all enrolled participants included in the analysis?		Yes
4.4 Were participants with missing data handled appropriately?		No information
4.5 Was selection of predictors based on univariable analysis avoided?		Yes
4.6 Were complexities in the data (e.g. censoring, competing risks, sampling of controls) accounted for appropriately?		Yes
4.7 Were relevant model performance measures evaluated appropriately?		Yes
4.8 Were model overfitting and optimism in model performance accounted for?		Not applicable
4.9 Do predictors and their assigned weights in the final model correspond to the results from multivariable analysis?		Not applicable
**Risk of bias introduced by the analysis**	**RISK:** (low/ high/ unclear)	Unclear
Rationale of bias rating: Did not report the missing data		

Pirattanakorn et al.

DOMAIN 1: Participants		
**A. Risk of Bias**		
1.1 Were appropriate data sources used, e.g. cohort, RCT or nested case-control study data?		Yes
1.2 Were all inclusions and exclusions of participants appropriate?		No
**Risk of bias introduced by selection of participants**	**RISK:** (low/ high/ unclear)	High
Rationale of bias rating: Some ICU patients were included in analysis		
**B. Applicability**		
**Concern that the included participants and setting do not match the review question**	**CONCERN:** (low/ high/ unclear)	High
Rationale of applicability rating: Some ICU patients were included in analysis		

DOMAIN 2: Predictors		
**A. Risk of Bias**		
2.1 Were predictors defined and assessed in a similar way for all participants?		Yes
2.2 Were predictor assessments made without knowledge of outcome data?		Yes
2.3 Are all predictors available at the time the model is intended to be used?		Yes
**Risk of bias introduced by predictors or their assessment**	**RISK:** (low/ high/ unclear)	Low
Rationale of bias rating: Low risk of bias		
**B. Applicability**		
Concern that the definition, assessment or timing of predictors in the model do not match the review question	**CONCERN:** (low/ high/ unclear)	Low
Rationale of applicability rating: Match the review question		

DOMAIN 3: Outcome		
**A. Risk of Bias**		
3.1 Was the outcome determined appropriately?		Yes
3.2 Was a pre-specified or standard outcome definition used?		Yes
3.3 Were predictors excluded from the outcome definition?		Yes
3.4 Was the outcome defined and determined in a similar way for all participants?		Yes
3.5 Was the outcome determined without knowledge of predictor information?		Yes
3.6 Was the time interval between predictor assessment and outcome determination appropriate?		Unclear
**Risk of bias introduced by the outcome or its determination**	**RISK:** (low/ high/ unclear)	Unclear
Rationale of bias rating: Scores were not calculated at hospital admission		
**B. Applicability**		
**Concern that the outcome, its definition, timing or determination do not match the review question**	**CONCERN:** (low/ high/ unclear)	High
Rationale of applicability rating: Scores were not calculated at hospital admission		

<table>
<tr><th colspan="3">DOMAIN 4: Analysis</th></tr>
<tr><th colspan="3">Risk of Bias</th></tr>
<tr><td colspan="2">4.1 Were there a reasonable number of participants with the outcome?</td><td>Yes</td></tr>
<tr><td colspan="2">4.2 Were continuous and categorical predictors handled appropriately?</td><td>Yes</td></tr>
<tr><td colspan="2">4.3 Were all enrolled participants included in the analysis?</td><td>Yes</td></tr>
<tr><td colspan="2">4.4 Were participants with missing data handled appropriately?</td><td>Yes</td></tr>
<tr><td colspan="2">4.5 Was selection of predictors based on univariable analysis avoided?</td><td>Yes</td></tr>
<tr><td colspan="2">4.6 Were complexities in the data (e.g. censoring, competing risks, sampling of controls) accounted for appropriately?</td><td>Yes</td></tr>
<tr><td colspan="2">4.7 Were relevant model performance measures evaluated appropriately?</td><td>Yes</td></tr>
<tr><td colspan="2">4.8 Were model overfitting and optimism in model performance accounted for?</td><td>Not applicable</td></tr>
<tr><td colspan="2">4.9 Do predictors and their assigned weights in the final model correspond to the results from multivariable analysis?</td><td>Not applicable</td></tr>
<tr><td>Risk of bias introduced by the analysis</td><td>RISK:
(low/ high/ unclear)</td><td>Low</td></tr>
<tr><td colspan="3">Rationale of bias rating:
Low risk of bias</td></tr>
</table>

Phungoen et al.

DOMAIN 1: Participants		
**A. Risk of Bias**		
1.1 Were appropriate data sources used, e.g. cohort, RCT or nested case-control study data?		Yes
1.2 Were all inclusions and exclusions of participants appropriate?		No
**Risk of bias introduced by selection of participants**	**RISK:** (low/ high/ unclear)	High
Rationale of bias rating: Retrospective cohort study		
**B. Applicability**		
**Concern that the included participants and setting do not match the review question**	**CONCERN:** (low/ high/ unclear)	Low
Rationale of applicability rating: Match the review question		

DOMAIN 2: Predictors		
**A. Risk of Bias**		
2.1 Were predictors defined and assessed in a similar way for all participants?		Yes
2.2 Were predictor assessments made without knowledge of outcome data?		Yes
2.3 Are all predictors available at the time the model is intended to be used?		Yes
**Risk of bias introduced by predictors or their assessment**	**RISK:** (low/ high/ unclear)	Low
Rationale of bias rating: Low risk of bias		
**B. Applicability**		
Concern that the definition, assessment or timing of predictors in the model do not match the review question	**CONCERN:** (low/ high/ unclear)	Low
Rationale of applicability rating: Match the review question		

DOMAIN 3: Outcome		
**A. Risk of Bias**		
3.1 Was the outcome determined appropriately?		Yes
3.2 Was a pre-specified or standard outcome definition used?		Yes
3.3 Were predictors excluded from the outcome definition?		Yes
3.4 Was the outcome defined and determined in a similar way for all participants?		Yes
3.5 Was the outcome determined without knowledge of predictor information?		Yes
3.6 Was the time interval between predictor assessment and outcome determination appropriate?		Yes
**Risk of bias introduced by the outcome or its determination**	**RISK:** (low/ high/ unclear)	Low
Rationale of bias rating: Low risk of bias		
**B. Applicability**		
**Concern that the outcome, its definition, timing or determination do not match the review question**	**CONCERN:** (low/ high/ unclear)	Low
Rationale of applicability rating: Match the review question		

DOMAIN 4: Analysis		
**Risk of Bias**		
4.1 Were there a reasonable number of participants with the outcome?		Yes
4.2 Were continuous and categorical predictors handled appropriately?		Yes
4.3 Were all enrolled participants included in the analysis?		Yes
4.4 Were participants with missing data handled appropriately?		No
4.5 Was selection of predictors based on univariable analysis avoided?		Yes
4.6 Were complexities in the data (e.g. censoring, competing risks, sampling of controls) accounted for appropriately?		Yes
4.7 Were relevant model performance measures evaluated appropriately?		Yes
4.8 Were model overfitting and optimism in model performance accounted for?		Not applicable
4.9 Do predictors and their assigned weights in the final model correspond to the results from multivariable analysis?		Not applicable
**Risk of bias introduced by the analysis**	**RISK:** (low/ high/ unclear)	High
Rationale of bias rating:<br>Patients with missing value were excluded		

Ruangsomboon et al.

DOMAIN 1: Participants		
**A. Risk of Bias**		
1.1 Were appropriate data sources used, e.g. cohort, RCT or nested case-control study data?		Yes
1.2 Were all inclusions and exclusions of participants appropriate?		Yes
**Risk of bias introduced by selection of participants**	**RISK:** (low/ high/ unclear)	Low
Rationale of bias rating: Low risk of bias		
**B. Applicability**		
**Concern that the included participants and setting do not match the review question**	**CONCERN:** (low/ high/ unclear)	Low
Rationale of applicability rating: Match the review question		

DOMAIN 2: Predictors		
**A. Risk of Bias**		
2.1 Were predictors defined and assessed in a similar way for all participants?		Yes
2.2 Were predictor assessments made without knowledge of outcome data?		Yes
2.3 Are all predictors available at the time the model is intended to be used?		Yes
**Risk of bias introduced by predictors or their assessment**	**RISK:** (low/ high/ unclear)	Low
Rationale of bias rating: Low risk of bias		
**B. Applicability**		
Concern that the definition, assessment or timing of predictors in the model do not match the review question	**CONCERN:** (low/ high/ unclear)	Low
Rationale of applicability rating: Match the review question		

DOMAIN 3: Outcome		
**A. Risk of Bias**		
3.1 Was the outcome determined appropriately?		Yes
3.2 Was a pre-specified or standard outcome definition used?		Yes
3.3 Were predictors excluded from the outcome definition?		Yes
3.4 Was the outcome defined and determined in a similar way for all participants?		Yes
3.5 Was the outcome determined without knowledge of predictor information?		Yes
3.6 Was the time interval between predictor assessment and outcome determination appropriate?		Yes
**Risk of bias introduced by the outcome or its determination**	**RISK:** (low/ high/ unclear)	Low
Rationale of bias rating: Low risk of bias		
**B. Applicability**		
**Concern that the outcome, its definition, timing or determination do not match the review question**	**CONCERN:** (low/ high/ unclear)	Low
Rationale of applicability rating: Match the review question		

DOMAIN 4: Analysis		
**Risk of Bias**		
4.1 Were there a reasonable number of participants with the outcome?		Yes
4.2 Were continuous and categorical predictors handled appropriately?		Yes
4.3 Were all enrolled participants included in the analysis?		Yes
4.4 Were participants with missing data handled appropriately?		Yes
4.5 Was selection of predictors based on univariable analysis avoided?		Yes
4.6 Were complexities in the data (e.g. censoring, competing risks, sampling of controls) accounted for appropriately?		Yes
4.7 Were relevant model performance measures evaluated appropriately?		Yes
4.8 Were model overfitting and optimism in model performance accounted for?		Not applicable
4.9 Do predictors and their assigned weights in the final model correspond to the results from multivariable analysis?		Not applicable
**Risk of bias introduced by the analysis**	**RISK:** (low/ high/ unclear)	Low
Rationale of bias rating: Low risk of bias		

Wattanasit et al.

DOMAIN 1: Participants		
**A. Risk of Bias**		
1.1 Were appropriate data sources used, e.g. cohort, RCT or nested case-control study data?		No
1.2 Were all inclusions and exclusions of participants appropriate?		Yes
**Risk of bias introduced by selection of participants**	**RISK:** (low/ high/ unclear)	High
Rationale of bias rating: Data was from retrospective cohort study		
**B. Applicability**		
**Concern that the included participants and setting do not match the review question**	**CONCERN:** (low/ high/ unclear)	Low
Rationale of applicability rating: Match the review question		

DOMAIN 2: Predictors		
**A. Risk of Bias**		
2.1 Were predictors defined and assessed in a similar way for all participants?		Yes
2.2 Were predictor assessments made without knowledge of outcome data?		Yes
2.3 Are all predictors available at the time the model is intended to be used?		Yes
**Risk of bias introduced by predictors or their assessment**	**RISK:** (low/ high/ unclear)	Low
Rationale of bias rating: Low risk of bias		
**B. Applicability**		
Concern that the definition, assessment or timing of predictors in the model do not match the review question	**CONCERN:** (low/ high/ unclear)	Low
Rationale of applicability rating: Match the review question		

DOMAIN 3: Outcome		
**A. Risk of Bias**		
3.1 Was the outcome determined appropriately?		Yes
3.2 Was a pre-specified or standard outcome definition used?		Yes
3.3 Were predictors excluded from the outcome definition?		Yes
3.4 Was the outcome defined and determined in a similar way for all participants?		Yes
3.5 Was the outcome determined without knowledge of predictor information?		Yes
3.6 Was the time interval between predictor assessment and outcome determination appropriate?		Low
**Risk of bias introduced by the outcome or its determination**	**RISK:** (low/ high/ unclear)	Yes
Rationale of bias rating: Low risk of bias		
**B. Applicability**		
**Concern that the outcome, its definition, timing or determination do not match the review question**	**CONCERN:** (low/ high/ unclear)	Low
Rationale of applicability rating: Match the review question		

DOMAIN 4: Analysis		
**Risk of Bias**		
4.1 Were there a reasonable number of participants with the outcome?		Yes
4.2 Were continuous and categorical predictors handled appropriately?		Yes
4.3 Were all enrolled participants included in the analysis?		Yes
4.4 Were participants with missing data handled appropriately?		Yes
4.5 Was selection of predictors based on univariable analysis avoided?		Yes
4.6 Were complexities in the data (e.g. censoring, competing risks, sampling of controls) accounted for appropriately?		Yes
4.7 Were relevant model performance measures evaluated appropriately?		Yes
4.8 Were model overfitting and optimism in model performance accounted for?		Not applicable
4.9 Do predictors and their assigned weights in the final model correspond to the results from multivariable analysis?		Not applicable
**Risk of bias introduced by the analysis**	**RISK:**<br>(low/ high/ unclear)	Low
Rationale of bias rating:<br>Low risk of bias		

Oduncu et al.

DOMAIN 1: Participants		
**A. Risk of Bias**		
1.1 Were appropriate data sources used, e.g. cohort, RCT or nested case-control study data?		Yes
1.2 Were all inclusions and exclusions of participants appropriate?		Yes
**Risk of bias introduced by selection of participants**	**RISK:** (low/ high/ unclear)	Low
Rationale of bias rating: Low risk of bias		
**B. Applicability**		
**Concern that the included participants and setting do not match the review question**	**CONCERN:** (low/ high/ unclear)	Low
Rationale of applicability rating: Match the review question		

DOMAIN 2: Predictors		
**A. Risk of Bias**		
2.1 Were predictors defined and assessed in a similar way for all participants?		Yes
2.2 Were predictor assessments made without knowledge of outcome data?		Yes
2.3 Are all predictors available at the time the model is intended to be used?		Yes
**Risk of bias introduced by predictors or their assessment**	**RISK:** (low/ high/ unclear)	Low
Rationale of bias rating: Low risk of bias		
**B. Applicability**		
Concern that the definition, assessment or timing of predictors in the model do not match the review question	**CONCERN:** (low/ high/ unclear)	Low
Rationale of applicability rating: Match the review question		

DOMAIN 3: Outcome		
**A. Risk of Bias**		
3.1 Was the outcome determined appropriately?		Yes
3.2 Was a pre-specified or standard outcome definition used?		Yes
3.3 Were predictors excluded from the outcome definition?		Yes
3.4 Was the outcome defined and determined in a similar way for all participants?		Yes
3.5 Was the outcome determined without knowledge of predictor information?		Yes
3.6 Was the time interval between predictor assessment and outcome determination appropriate?		Yes
**Risk of bias introduced by the outcome or its determination**	**RISK:** (low/ high/ unclear)	Low
Rationale of bias rating: Low risk of bias		
**B. Applicability**		
**Concern that the outcome, its definition, timing or determination do not match the review question**	**CONCERN:** (low/ high/ unclear)	Low
Rationale of applicability rating: Match the review question		

DOMAIN 4: Analysis		
**Risk of Bias**		
4.1 Were there a reasonable number of participants with the outcome?		Yes
4.2 Were continuous and categorical predictors handled appropriately?		Yes
4.3 Were all enrolled participants included in the analysis?		Yes
4.4 Were participants with missing data handled appropriately?		Yes
4.5 Was selection of predictors based on univariable analysis avoided?		Yes
4.6 Were complexities in the data (e.g. censoring, competing risks, sampling of controls) accounted for appropriately?		Yes
4.7 Were relevant model performance measures evaluated appropriately?		Yes
4.8 Were model overfitting and optimism in model performance accounted for?		Not applicable
4.9 Do predictors and their assigned weights in the final model correspond to the results from multivariable analysis?		Not applicable
**Risk of bias introduced by the analysis**	**RISK:** (low/ high/ unclear)	Low
Rationale of bias rating:<br>Low risk of bias		
