# Supplementary material for: National Early Warning Score Does Not Accurately Predict Mortality for Patients With Infection Outside the Intensive Care Unit: A Systematic Review and Meta-Analysis
Source: Front Med (Lausanne). 2021 Jul 15;8:704358. doi: 10.3389/fmed.2021.704358 (PMC8319382; doi:10.3389/fmed.2021.704358)
Supplement: Supplementary file 5 [file Data_Sheet_5.docx]

| First author (Year) | Oduncu 2021 |
| --- | --- |
| Study type | Prospective, single-center study |
| Country and setting | Turkey, emergency department |
| Participants Number Age Male% | Adult patients with suspected infections  463  63±18  59.2 |
| Identification criteria | Antibiotic and body fluid culture |
| Missing value  Score calculation time | No missing value  At emergency department admission |

| First author (Year) | Almutary 2020 |
| --- | --- |
| Study type | Retrospective, single-center study |
| Country and setting | Saudi Arabia, emergency department |
| Participants Number Age Male% | Adult patients with suspected sepsis  444  58.7±23.3  48.2 |
| Identification criteria | NR |
| Missing value  Score calculation time | NR  At emergency department admission |

| First author (Year) | Pairattanakorn 2020 |
| --- | --- |
| Study type | Prospective, single-center study |
| Country and setting | Thailand, all wards |
| Participants Number Age Male% | Adult patients with suspected sepsis  409  65.74±17.84  51.3 |
| Identification criteria | Blood culture |
| Missing value  Score calculation time | No missing value  At the time of blood draw or within 6 hours prior to blood culture |

| First author (Year) | Phungoen 2020 |
| --- | --- |
| Study type | Retrospective, single-center study |
| Country and setting | Thailand, emergency department |
| Participants Number Age Male% | Adult patients with suspected sepsis  8177  62 (median)  52.3 |
| Identification criteria | Blood culture and antibiotic |
| Missing value  Score calculation time | Patients with missing value were excluded  At ED admission |

| First author (Year) | Ruangsomboon 2020 |
| --- | --- |
| Study type | Retrospective, single-center study |
| Country and setting | Thailand, emergency department |
| Participants Number Age Male% | Adult patients with suspected sepsis  1622  72.6±15.4  48.9 |
| Identification criteria | Sepsis-3 definition |
| Missing value  Score calculation time | No missing value  At ED admission |

| First author (Year) | Wattanasit 2020 |
| --- | --- |
| Study type | Retrospective, single-center study |
| Country and setting | Thailand, emergency department |
| Participants Number Age Male% | Adult patients with suspected infection  777  67 (median)  54.3 |
| Identification criteria | Receiving intravenous antibiotics with the collection of any cultures |
| Missing value  Score calculation time | No missing value  First encountered in the ED |

| First author (Year) | Saeed 2019 |
| --- | --- |
| Study type | Retrospective, multicenter study |
| Country and setting | UK, France, Italy, Sweden and Spain, emergency department |
| Participants Number Age Male% | Adult patients (≥ 18 years) with clinical suspicion of infection  1175  63.3±20.9  50.4 |
| Identification criteria | Main presenting symptoms, vital signs, blood culture request or laboratory findings |
| Missing value  Score calculation time | Patients with missing value were excluded  At emergency department arrival |

| First author (Year) | Pong 2019 |
| --- | --- |
| Study type | Retrospective, single-center study |
| Country and setting | Singapore, emergency department |
| Participants Number Age Male% | Adult patients (aged 18 years and above) with suspected sepsis  364  Survivors: 65.7±16.4; Non-survivors: 72.8±15.0  49.1 |
| Identification criteria | Sepsis was defined as two or more SIRS criteria, concern for infection |
| Missing value  Score calculation time | Median interpolation  First recorded value obtained upon patient presentation |

| First author (Year) | Melhammar 2019 |
| --- | --- |
| Study type | Retrospective, single-center study |
| Country and setting | Sweden, emergency department |
| Participants Number Age Male% | Adult patients had a suspected infection and at least one of SIRS criteria at presentation  526  58 (median)  55.5 |
| Identification criteria | Clinical identification |
| Missing value  Score calculation time | Patients with missing value were excluded  At emergency department arrival |

| First author (Year) | Fernando 2019 |
| --- | --- |
| Study type | Retrospective, multicenter study |
| Country and setting | Canada, general ward |
| Participants  Number Age Male% | Adult patients with suspected infection, and fulfilled rapid response team calling criteria  1708  NR  NR |
| Identification criteria | Infection was defined by antibiotics use and body fluid cultures |
| Missing value  Score calculation time | Patients with missing value were excluded  At hospital admission |

| First author (Year) | Chiew 2019 |
| --- | --- |
| Study type | Retrospective, single-center study |
| Country and setting | Singapore, emergency department |
| Participants  Number Age Male% | Adult patients with suspected sepsis  214  Survivors: 66 (median); Non-survivors: 76 (median)  50 |
| Identification criteria | Sepsis-1 criteria |
| Missing value  Score calculation time | NR  At admission |

| First author (Year) | Castillo 2019 |
| --- | --- |
| Study type | Prospective, multicenter study |
| Country and setting | Spain, emergency department |
| Participants Number Age Male% | Adult patients with suspected infection  684  65.1±19.6  53.5 |
| Identification criteria | Infection was identified by vital signs, main presenting symptoms, the blood culture, or laboratory findings |
| Missing value  Score calculation time | No missing value  At emergency department admission |

| First author (Year) | Brink 2019 |
| --- | --- |
| Study type | Retrospective, single-center study |
| Country and setting | Netherlands, emergency department |
| Participants Number Age Male% | Patients with suspected sepsis  8204  57 (41-68; median with IQR)  55.8 |
| Identification criteria | Intravenous antibiotic therapy or bacterial culture |
| Missing value  Score calculation time | Imputed five times using non-missing parameters  NR |

| First author (Year) | Lynn 2018 |
| --- | --- |
| Study type | Prospective, single-center study |
| Country and setting | Myanmar, general ward |
| Participants Number Age Male% | Patients admitted to the adult medical ward with sepsis  120  47 (28-63; median with IQR)  56.7 |
| Identification criteria | Sepsis-3 criteria |
| Missing value  Score calculation time | NR  At admission |

| First author (Year) | Szakmany 2018 |
| --- | --- |
| Study type | Prospective, multicenter study |
| Country and setting | UK, emergency department |
| Participants Number Age Male% | Adult patients with suspected or proven infection  380  74 (61-83; median with IQR)  47.3 |
| Identification criteria | Clinical identification |
| Missing value  Score calculation time | No missing value  At emergency department entry |

| First author (Year) | Redfern 2018 |
| --- | --- |
| Study type | Retrospective, single-center study |
| Country and setting | UK, general ward |
| Participants Number Age Male% | Adult patients with suspected or proven infection  44647  65±22  47.5 |
| Identification criteria | ICD-10 code |
| Missing value  Score calculation time | NR  NR |

| First author (Year) | Camm 2018 |
| --- | --- |
| Study type | Retrospective, single-center study |
| Country and setting | UK, emergency department |
| Participants Number Age Male% | Adult patients with suspected infection  316  74.5±16.6  47.5 |
| Identification criteria | Clinical identification |
| Missing value  Score calculation time | Missing value were excluded in analysis  NR |

| First author (Year) | Groot 2017 |
| --- | --- |
| Study type | Retrospective, multicenter study |
| Country and setting | Netherlands, emergency department |
| Participants Number Age Male% | Adult patients with suspected infection  2280  61.1±17.0  57.7 |
| Identification criteria | Hospital coding system |
| Missing value  Score calculation time | Missing data were assumed to be normal  At admission |

| First author (Year) | Goulden 2017 |
| --- | --- |
| Study type | Retrospective, single-center study |
| Country and setting | UK, emergency department |
| Participants Number Age Male% | Adult patients with suspected sepsis  1818  68±19  49 |
| Identification criteria | Clinical identification |
| Missing value  Score calculation time | Multiple imputation  At admission |

| First author (Year) | Churpek 2017 |
| --- | --- |
| Study type | Retrospective, single-center study |
| Country and setting | USA, emergency department and general ward |
| Participants Number Age Male% | Adult patients with suspected infection  30677  58±18  47 |
| Identification criteria | Any culture order followed by an intravenous antibiotic within 72 hours or an intravenous antimicrobial followed by a culture order within 24 hours |
| Missing value  Score calculation time | Previous values were pulled forward if they were missing, and if no previous values were available, a median value was imputed  NR |

| First author (Year) | Corfield 2014 |
| --- | --- |
| Study type | Retrospective, multicenter study |
| Country and setting | UK, emergency department |
| Participants Number Age Male% | Adult patients with suspected sepsis  2003  72 (median)  47 |
| Identification criteria | Sepsis-1 criteria |
| Missing value  Score calculation time | Patients with missing value were excluded  NR |
