# Supplementary figures and images for: National Early Warning Score Does Not Accurately Predict Mortality for Patients With Infection Outside the Intensive Care Unit: A Systematic Review and Meta-Analysis
Source: Front Med (Lausanne). 2021 Jul 15;8:704358. doi: 10.3389/fmed.2021.704358 (PMC8319382; doi:10.3389/fmed.2021.704358)

Deeks' Funnel Plot Asymmetry Test  
pvalue = 0.10

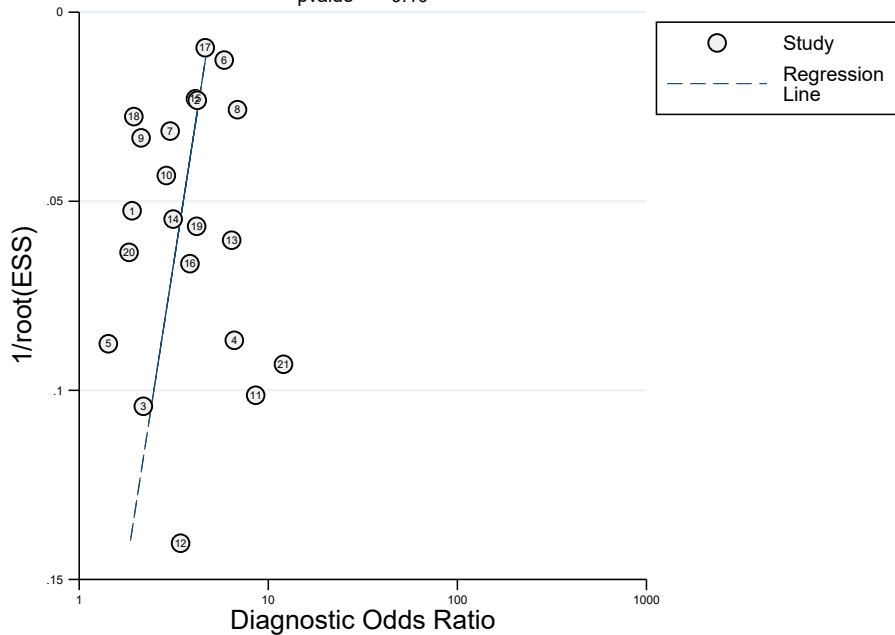

Supplement: Supplementary file 6 [file Data_Sheet_6.pdf]
